# Compensatory phenolic induction dynamics in aspen after aphid infestation

**DOI:** 10.1038/s41598-022-13225-x

**Published:** 2022-06-10

**Authors:** Rajarshi Kumar Gaur, Ilka Nacif de Abreu, Benedicte Riber Albrectsen

**Affiliations:** 1 Department of Plant Physiology, Umeå Plant Science Centre, 90187 Umeå, Sweden; 2 Department of Biotechnology, Deen Dayal Upadhyaya Gorakhpur University, Gorakhpur, Uttar Pradesh 273009 India

**Keywords:** Biochemistry, Plant sciences

## Abstract

Condensed tannins (CTs) are polyphenolics and part of the total phenolic (TP) pool that shape resistance in aspen (*Populus tremula*). CTs are negatively associated with pathogens, but their resistance properties against herbivores are less understood. CTs shape resistance to pathogens and chewing herbivores and could also shape resistance to aphids. Being chemical pools that are highly variable it can further be questioned whether CT-shaped resistance is better described by constitutive levels, by the induced response potential, or by both. Here, aspen genotypes were propagated and selected to represent a range of inherent abilities to produce and store foliar CTs; the plantlets were then exposed to *Chaitophorus* aphid infestation and to mechanical (leaf rupture) damage, and the relative abundance of constitutive and induced CTs was related to aphid fitness parameters. As expected, aphid fecundity was negatively related to CT-concentrations of the aphid infested plants although more consistently related to TPs. While TPs increased in response to damage, CT induction was generally low and it even dropped below constitutive levels in more CT-rich genotypes, suggesting that constitutive CTs are more relevant measurements of resistance compared to induced CT-levels. Relating CT and TP dynamics with phenolic low molecular compounds further suggested that catechin (the building block of CTs) increased in response to aphid damage in amounts that correlated negatively with CT-induction and positively with constitutive CT-levels and aphid fecundity. Our study portrays dynamic phenolic responses to two kinds of damage detailed for major phenylpropanoid classes and suggests that the ability of a genotype to produce and store CTs may be a measurement of resistance, caused by other, more reactive, phenolic compounds such as catechin. Rupture damage however appeared to induce catechin levels oppositely supporting that CTs may respond differently to different kinds of damage.

## Introduction

A central question in ecology is how plants withstand dangers in their environment, and phenolic compounds are frequently explored to address plant resistance properties^1–3^. As plants adapted to the terrestrial environment, a diversification of plant and associated arthropod herbivore taxon took place^4^, which was paralleled by the evolution and diversification of the phenylpropanoid biosynthetic pathway^5,6^. The wealth of phenolic compounds from this pathway that we find in plants today support many protective and structural functions in plants’ terrestrial lifestyle such as tissue-strengthening lignins, photo-protecting anthocyanidins, and toxic defence phenolics often neutralised by glycoside associations.

Several biosynthetic and heritable aspects of these compound groups are well characterised. The phenylpropanoid pathway to plant phenolic compounds is reticulate with biosynthetic cross-links between substrates and enzymes that connect side branches^7–10^, and competition for substrate may happen throughout the pathway. Phenylalanine for example both acts as a protein building block and as substrate of the phenylpropanoid pathway; thus, linking general and specialised metabolism together^5,11^. Salicinoid phenolic glycosides (SPGs, one of two major defence phenolic classes) belong to a part of the biosynthetic pathway, which is less described, and here enzyme competition for salicortin substrate has been suggested to explain genotypic specificity in SPG-profiles of European aspen (*Populus tremula*)^12^. Polyphenolic condensed tannins (CTs, the second major class of defense phenolic classes) are built from flavan-3-ol (catechin) units and in aspen vary considerably in concentration for example in response to anthropogenic nitrogen addition^13^, age and ontogeny^14^. Aspen genotypes’ ability to synthetise, accumulate and store CTs is heritable^15^ allowing for relative characterization of CT producers within a population^16^. Flavan-3-ols that the CTs are made from are products of a branch pathway of anthocyanin biosynthesis^17^ and polymerization to CTs appears to be a non-enzymatic process during which proanthocyanidin starter and extension dimers assemble in the vacuole^18^, however it is unknown whether *in planta* degradation of CTs can also be a spontaneous process. CTs bind proteins and are known for their antimicrobial^19–22^, and deterrent properties to chewing herbivores^23–27^.

Upon damage, defence compounds are induced and the more we understand plant metabolism under stress, the more we know that the stressed plant metabolome undergoes dynamic coherent mass events of which we capture only snap shots of a multitude associated single compounds, compound classes, and entire pathways^28^. In naturally varying stands of trees, correlative relationships between defence compounds and antagonists are often incomplete^29–31^, and although phenolic chemistry and genotype may explain resistance traits for particular biological associations, community associations with phenolic profiles are often weak^15^. Random events may partly explain the imperfect relationships, but inducibility also makes plant chemical profiles moving targets^32^, prompting the question of whether constitutive defence levels correlate with induced defence responses, or if either of the two better explain resistance properties.

Ideas about optimal allocation of resources have suggested that plants’ defence metabolic investments are balanced between needs for growth and defence^11,33,34^. Compensatory investment into defence pools directed towards antagonist groups might also be expected, however while CTs and SPGs may balance one another at the population scale^14^, there seems to be no simple trade-off between investment into CTs and SPGs within individual trees^15,19^, nor when the genotypes are grouped into behaviours that characterise tannin extreme phenotypes^35^. The multiple routes to many phenolic compound pools, with biosynthetic cross-links between substrates and enzymes that connect side branches of the pathway^7–10^, may complicate our ability to detect interrelationships between specific phenolic pools, and our insight into how biotic stress may translate into biosynthetic priorities of the phenylpropanoid pathway remain limited.

Induction behaviours in response to antagonists provide specific clues about phenol metabolic priorities. Infection by the biotrophic rust (*Melampsora larici-populina*) in CT-rich poplar hybrids initiates the hormonal Salicylic Acid (SA) signalling pathway, causing an upregulation of the antioxidative flavan-3-ol biosynthesis, which improves the plants’ tolerance to reactive oxygen species (ROS)^20,21^. Aphid infestations share many similarities with those of pathogen infections. In *Arabidopsis*, in their search for phloem sap, aphids also induce the SA-hormonal pathway, generate ROS (such as H_2_O_2_)^36–38^_,_ and cause accumulation of defence phenolics^39^. However, phenolic defensive interactions with piercing–sucking herbivores such as aphids have rarely been detailed in aspen^40^.

Aspen trees (*Populus tremula*) are outcrossing and genetically diverse keystone species of the northern hemisphere^41,42^, that like North American aspen (*Populus tremuloides*), produce CT and SPG defence phenolic compounds in leaves, bark, and roots^3,23,25,43,44^ that considerably contribute to the pool of total phenolics (TPs)^13^. In this study we used Aspen genotypes that represented a range of CT genotypes from the Swedish Aspen (SwAsp) collection^16,45^, to study the relationship between genotype specific constitutive CT concentrations and induced responses after two kinds of damage: infestation by *Chaitophorus* aphids and mechanical rupture. We show that aphid fecundity is negatively related to both TPs and CTs but not to specific SPGs. We also show how CT induction negatively associated with constitutive CT levels measured in control plants, in contrast to the association with TPs that correlated with the induction of antioxidant catechin flavan-3-ols. This suggests that it may be the catechin building blocks of CTs that provide the active defence mechanism against aphids.

## Materials and methods

### Plant materials

SwAsp genotypes 5, 23, 36, 47, 51 and 72 of *P. tremula*^45^ were propagated from tissue culture available at Umeå Plant Science Centre, Sweden and maintained in the SLU greenhouse, (room temperature, 18:6 L:D) Umeå, Sweden until 1 week before the experiment was initiated in the climate chamber (~ 18 °C, 18:6 L:D, RH ~ 75%). Plants were moved to the climate chamber one week before each experiment and plant height was measured in cm (from soil to apical meristem of longest shoot) and numbers of leaves (fully expanded) were counted. The growth measurements were repeated as soon as the experiment had been terminated.

In two balanced experiments, aspen genotypes were subjected to the following treatments: leaf rupture, aphid infestation, and untreated controls. Three replicates were used per treatment, genotype, and experiment. The experiments took place in 2007 on 3–4 months old plants approximately 1 month apart: November 9th to November 16th ~ Exp1 (using five genotypes), and December 11th to December 18th ~ Exp2 (using six genotypes). During each experiment, plants (ranging from 40 to 120 cm in height across genotypes, experiments, and time points) grew in 5 l pots in mixed sand, peat, and loam (51:48:1). The pots were positioned in saucers and placed on the floor in a random pattern with > 80 cm between leaves from any pair of the plantlets, which also facilitated inspection of the trees without risk of transferring aphids among them.

Experimental research and field studies on plants including the collection of plant material, complied with relevant institutional, national, and international guidelines and legislation. The Aspen plantlets used in this study were propagated from the SwAsp collection of 106 natural varying European Aspen (*Populus tremula*) genotypes originated from twelve sites in Sweden up to 2000 km apart. Genotypes from the collection are kept in tissue culture at the Umeå Plant Science Centre. The SwAsp resource is publicly available through contact with the UPSC poplar transgenics facility https://www.upsc.se/. No licence no. or ethical requirements are needed to grow or transport SwAsp plantlets or trees within Sweden.

### Aphid material

Free-living specialist *Chaitophorus* aphids are commonly found on aspen and our culture was established from one colony as described in Ref.^46^. Twenty individual aphids belonging to three species were placed singly on 60 cm tall aspen trees to reproduce. One well performing line of aphids, was then chosen for the experiment. The line was preliminarily identified as *Chaitophorus populeti*. The images that should have confirmed the species identity after the termination of the experiment was however lost and consequently, we refer to the line of aphids that we used for this experiment only by their genus name.

### Treatments: rupture and aphid infestation

The most apical unfurled leaf was designated as leaf position one and progressively lower leaves were numbered sequentially. Due to phyllotaxis which follows a sequence of six leaves in *Populus* (Refs.^47,48^, we applied treatments to six sequential fully-expanded mature leaves (“local leaves” at leaf position numbers 17–12). The six leaves right above the treated leaves were then considered “systemic” as they were vascularly connected with the infested leaves^48^, and we harvested three of those leaves for chemical analyses (please see details below). To rupture leaves we used a dog bristle brush to punch minute holes into each leaf. The punch holes would cover the entire leaf surface with approximately ten holes per cm^2^ and a paper towel positioned under the surface during the treatment to protect the leaf from other damage. For the aphid treatment, six adult, apterous female aphids (*Chaitophorus* sp. tentatively determined to *C. populeti* Panzer, 1804) were positioned singly on each of the leaves. Several *Chaitophorus* aphids live on aspen^46^, however the only *Populus tremula* is the natural host for *C. populeti* in Sweden although it also used *P. nigra* and *P. alba* and their hybrids in Southern Europe. Although free living, when placed on leaves the aphids stayed and initiated their probing activity^41^. After placing the aphids on the plants, we carefully inspected potential movements on the plants for a couple of hours to secure that founder aphids stayed on their leaves and initiated probing. Thereafter plants were left to be inspected at 24 h intervals, and the numbers of nymphs and adult aphids where then counted daily on every infested leaf. The experiment had been terminated had winged forms started to emerge or if absence of founder aphids on the assigned leaves had been detected on any plant.

### Leaf chemistry

Before the start of each experiment, anthocyanin levels in leaves were measured non-destructively by ratio of absorbances at 530 nm (anthocyanin) and 931 nm (reference at Near Infra-Red range) with a CCM-200A instrument (Opti Sciences, Inc, USA) on three mature leaves at positions 17–15 (bottom) and positions 10–8 (top). Immediately after the experiment had been terminated (189 h after infestation and damage, the three middle “systemic” leaves (leaf positions 10–8) were harvested from each plant and flash frozen in liquid nitrogen, kept at − 80 °C until ground with a mortar and pestle while cooled in liquid nitrogen, and at all times kept at − 80 °C until chemical analyses. For all experimental plants the sampled leaf material was analysed for total phenolics and CT concentrations.

#### Total phenolics

50 mg (FW) homogenised leaf sample was extracted with 1.8 ml of methanol (80%, v/v). Samples were vortexed and kept at room temperature for 30 min and then centrifuged for 10 min at 14,000 rpm. Total soluble phenolic content was measured using the Folin-Ciocalteu assay^49^ and quantifications were calculated based on a chlorogenic acid standard curve (0–40 µg/µl). 200 μl of the supernatant was freeze-dried in a speedvac and kept frozen for later LC/MS-analyses.

#### Condensed tannins

About 20 mg (FW) of the leaf powder was suspended in 0.5 ml of Acetone (70% v/v, 1% ascorbic acid), allowed to stand for 1 h at room temperature, and centrifuged for 10 min at 14,000 rpm. The resulting pellet was extracted twice, and acetone extract was dried by speedvac. This purified extract was then used to determine soluble CTs, using the assay described by Ossipova et al.^50^ and quantifications were conducted by use of a standard curve of procyanidin (0–80 µg/µl, Merck 42157, Procyanidin B2).

#### Phenolic analysis by LC/MS

The phenolic profile was determined by UHPLC-TOF MS as described in Abreu et al.^49^. Before analysis, the stored frozen extracts were reconstituted with 20 μl of methanol and 20 μl of water. 2 μl of the reconstituted plant extracts were injected and separated on a C18 UPLC™ column (2.1 × 100 mm, 1.7 μm) and analysed with a LCT Premier TOF/ MS (all from Waters, Milford, MA, USA) in negative mode. MS files were processed by MassLynx 4.1 software package (Waters Corp.) by a targeted approach using an in-house phenolic database. The resulting peak areas were normalised according to an internal standard and sample weight.

### Statistical analyses

Constitutive phytochemical values were measured as genotype means in control plants, and this genotype mean was subtracted from the treatment induced level for every aphid infested plant of that genotype to provide the relevant level of induction. Statistical analyses were conducted in R (R Core Team version 3.6.3, 2019^51^), package lme4 version 1.1-21^52^ and rsq version 2.2^53^, and plotted with ggplot2^54^ incl. extensions Patchwork, Version 1.1.1^55^, and facto extra^56^.

### Studies involving animal subjects

No animal studies are presented in this manuscript.

### Studies involving human subjects

No human studies are presented in this manuscript.

### Inclusion of identifiable human data

No potentially identifiable human images or data is presented in this study.

## Results

### Greenhouse experiments were conducted on homogenous plant material

Tests for bias in the initial plant materials revealed no significant differences among plants selected for the two experiments (Height: *P* = 0.67, Leaf Production: *P* = 0.79), nor among the three treatment groups (Height: *P* = 0.85, Leaf Production: *P* = 0.72). However, as expected^15^, genotypic variation in untreated plants was detected for growth and chemical traits with SwAsp72 being taller, and SwAsp47 producing more leaves and having higher amounts of anthocyanins (Height: *P* < 0.0001, Leaf production: P < 0.01, and Anthocyanin levels: *P* < 0.0001, Supplementary Material File S1). In addition, although there were significant experimental effects (Supplementary Material File S2a–c), aphids on SwAsp36 and 51 reproduced more successfully compared to aphids on SwAsp47 and 72.

### Induced responses for CTs differed between treatments

**Figure 1:**
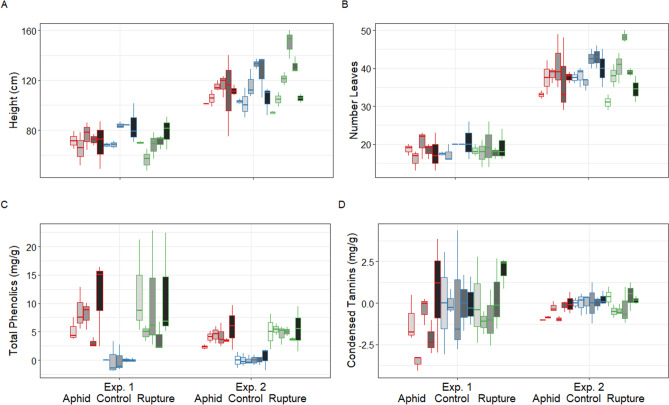
Boxplots of induced growth and defence phenotypes of aspen plantlets (grey coloured after genotype) after infestation by *Chaitophora* aphids (red frame), mechanical leaf rupture (green frame), and controls (blue frame). Responses include: (**A**) height; (**B**) leaf production; and foliar concentrations in mg/g FW of: (**C**) total phenolics; and (**D**) condensed tannins, during two experimental set-ups that took place on plantlets from the same batch one month apart. Horizontal lines in the boxes indicate the median and box boundaries 25 and 75 percentile, respectively. Vertical lines represent the range of values per tested unit (n = 3). Means ± s.e. are detailed in Table 1, together with test specifics.

To compare genotype induction strengths, response values after infestation and rupture were adjusted by subtraction from the control group genotype specific mean. Varied treatment effects were found for growth traits and phenolic traits in response to both aphid infestation and leaf rupture (Fig. 1A–D, Table 1). Height increase was most impaired by damage in Experiment 1 (Fig. 1A), whereas leaf production was most negatively affected by the damage treatments during Experiment 2 (Fig. 1B). However, all plants grew taller and produced leaves during both experiments; in no case was stunted growth absolute, suggesting that all experimental plants had spare metabolic resources to allocate. Total phenolics in leaves always increased in response to treatment when compared to control plants, and at rates defined by genotype identity and experiment (Fig. 1C, Table 1). CTs declined in response to aphid infestation (Fig. 1D), with greatest decrease values for the innately high CT genotypes SwAsp36 and 72 (Table 1)^16^. Although based on only three replicates per genotype and treatment, consistent responses to the two kinds of stress suggested specificity to treatment (Fig. 1, Table 1). A steady increase in total oxidative capacity (TPs) was indicated by high t-values for both treatments (Table 1, aphid infestation t = 3.21 ~ P < 0.0001 and leaf rupture t = 5.10 ~ P < 0.0001). CT-levels did not change as radically as TPs but our results did suggest a reduction in tannin levels in response to the aphid infestation (t = − 3.09, ~ P < 0.0001).

**Table 1:** Model summaries of growth and defence phenolic responses as a function of SwAsp genotype and treatment, using a mixed-effects linear regression model controlled for the effect of experimental setup.

Responses	Final leaf number*)		Final height		CTs induction*)		TPs induction*)	
**Models and effects**								
M0 ~ 1 + (1 | Exp)	**M0: AIC** = **− 72.7, BIC** = ** − 65.2**		M0: AIC = 751.1, BIC = 758.6		M0: AIC = 115.1, BIC = 122.6		M0: AIC = 68.5, BIC = 75.8	
M1 ~ 1 + G + T + ( 1 | Exp)	M1: AIC = 521.6, BIC = 546.6		**M1: AIC** = **736.2, BIC** = **716.0**		**M1: AIC** = **104.7, BIC** = **129.7**		**M1: AIC = − 16.5, BIC = 8.5**	
M2 ~ 1 + G* T + (1 |Exp)	M2: AIC = 529.8, BIC = 579.8		M2: AIC = 749.2, BIC = 799.2		M2: AIC = 117.8, BIC = 167.8		M2: AIC = − 3.5, BIC = 46.5	
Pseudo-R^2^ (total)	0.86		0.79		0.85		0.81	
Random effect (1 | Exp)	Df: 88; LRT: 167.64***		Df: 88; LRT: 116.92***		Df: 88; LRT: 149.58***		Df: 88; LRT: 82.91***	

	Mean ± s.e.	t-value	Mean ± s.e.	t-value	Mean ± s.e.	-value	Mean ± s.e.	t-value
Fixed effects intercept	3.28 ± 0.21	12.56**	89.17 ± 16.21	5.50*	3.1 ± 1.82	1.86^***n.s***^	9.78 ± 1.22	2.84***
Infestation			*** − ***4.95 ± 3.16	*** − ***1.56^***n.s***^	0.75 ± 1.09	*** − ***3.09***	1.45 ± 1.12	3.21***
Rupture			*** − ***0.94 ± 3.19	*** − ***0.29^***n.s***^	0.97 ± 1.11	*** − ***0.30^***n.s***^	1.79 ± 1.12	5.10***
SwAsp36			3.19 ± 4.28	0.75^***n.s***^	0.85 ± 1.14	*** − ***1.29^***n.s***^	0.85 ± 1.12	*** − ***1.42 ^***n.s***^
SwAsp47			17.11 ± 4.33	3.95***	0.88 ± 1.14	*** − ***1.00^***n.s***^	0.84 ± 1.12	*** − ***1.44 ^***n.s***^
SwAsp5			*** − ***10.14 ± 6.08	*** − ***1.67^***n.s***^	1.25 ± 1.20	1.19^***n.s***^	0.86 ± 1.16	*** − ***0.98 ^***n.s***^
SwAsp51			11.71 ± 4.34	2.70**	1.31 ± 1.14	2.06*	0.82 ± 1.12	1.77*
SwAsp72			5.75 ± 4.33	1.33^***n.s***^	1.03 ± 1.14	0.27^***n.s***^	1.25 ± 1.12	1.98*

Model comparison was used to select for the best model; models were selected as the more parsimonious when Akaike Criteria Index values (AIC) were two or more units lower than the second-best model. Model indication = G for genotype and T for treatment, (1|Exp) controlled for experimental set-up. Mo = Null model = intercept, M1 = two-factor model, M2 = two factor model with interaction. *log transformed response values to meet criteria on normally distributed residuals. Genotype and treatment comparisons are related to SwAsp23 and the control treatment responses embedded in the Fixed factor Intercept. Numbers indicate: Mean ± s.e., and t-values for fixed effects. Significance levels: Model significance***P < 0.0001; **P < 0.01; *P < 0.5; *n.s.* not significant. Sample size for each box = 3.

Chosen models are in bold, *) implies use of log transformed response values.

### Aphid reproduction positively correlated with growth and negatively with defence phenolics

**Figure 2:**
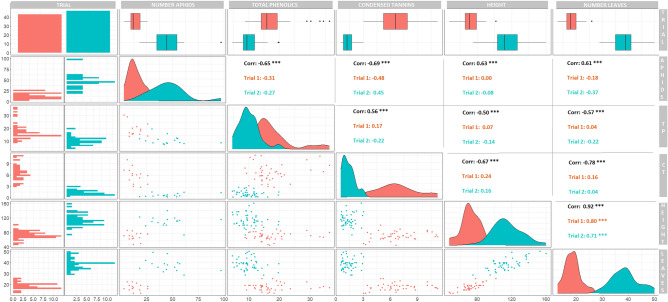
Scatterplot matrix displaying Pearson pairwise correlative relationships between plant growth and defence responses and aphid fecundity responses measured for the aspen plantlets of this study. Sample distribution plots follow the diagonal line, scatterplots of values are shown in the triangular space below the diagonal axis and Pearson’s correlation values for the same relationships are indicated in the upper triangle (N = 99). Aphids included both adults and nymphs on a plant, total phenolics (TPs), and condensed tannins (CTs) in mg/g FW, plant height in cm and number of leaves.

Across the two experimental set-ups, growth metrics (height and leaf production) were highly correlated (Fig. 2, Pearson’s r = 0.92), and negatively related to leaf content of TPs (− 0.50 < Pearson’s r < − 0.57) and to CTs (− 0.67 < Pearson’s r < − 0.78). Total phenolic measurements included CT concentrations and they were positively correlated (TPs and CTs: Pearson’s r = 0.56). Bi-modal distributions characterised the data (Fig. 2) following the two experimental set-ups that were conducted 1 month apart.

Aphids reproduced better on the more productive plants. The total number of aphids (offspring and surviving founders) positively correlated with growth parameters (0.61 < Pearson’s r < 0.63) and negatively with phenolic content (− 0.65 < Pearson’s r < − 0.69).

**Figure 3:**
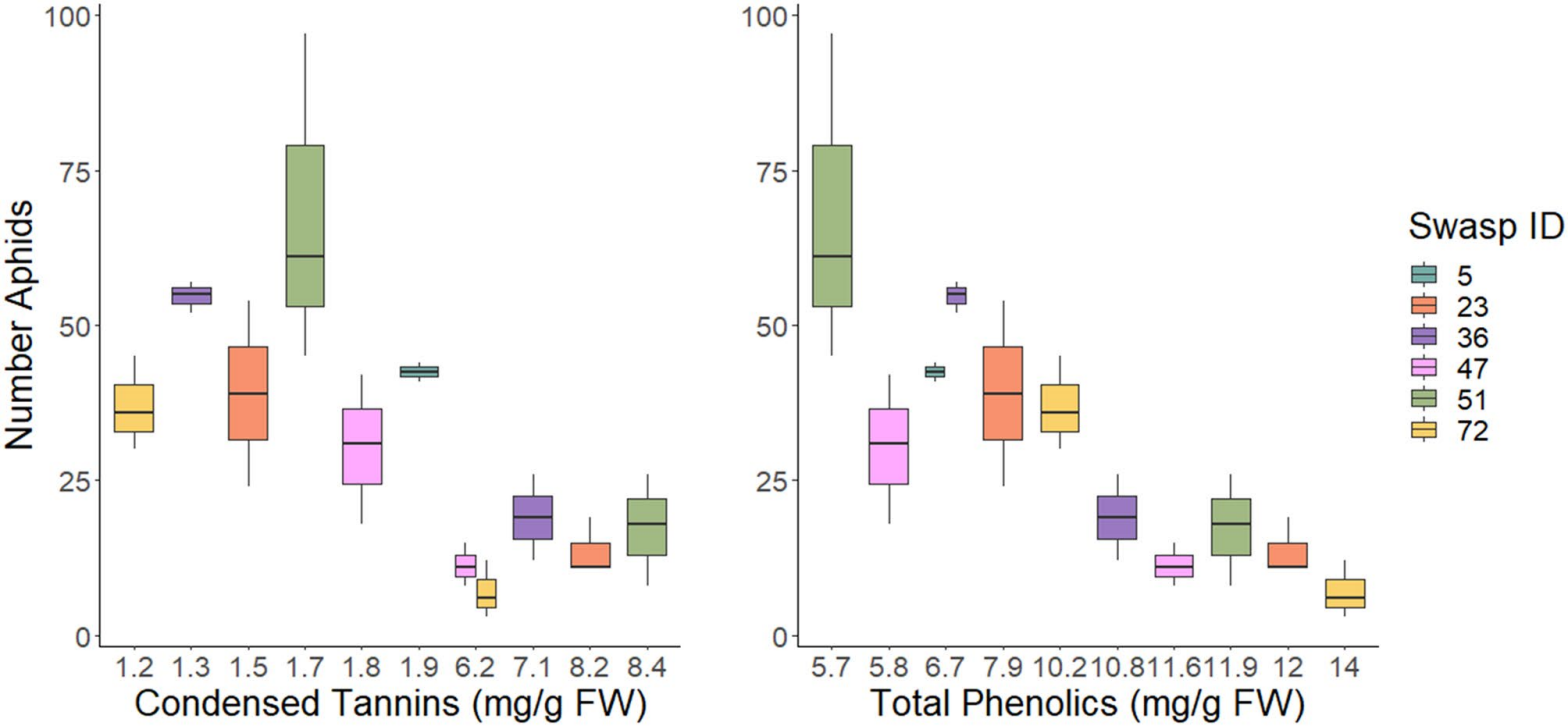
Boxplots highlighting the relationship between induced leaf phenolic groupings after aphid infestation and aphid population development. (left) Aphid population (both adults and nymphs) as a function of induced CTs, and (right) aphid population as an effect of induced TPs.

Survival of founder aphids could not be explained by phenolic measurements (Table 2), whereas reproduction was negatively associated with levels of TPs and CTs measured in untreated controls (Table 2) although less for CTs compared to TPs (Fig. 3). Foliar anthocyanin in the upper most red-coloured leaves also negatively associated with aphid reproduction (*P* = 0.058, Table 2), however, no strong aphid inhibitory effect was detected for any specific phenolic compounds (linear fits in Supplementary Material File S4).

**Table 2:** Aphid survival and fecundity.

Aphids	Phenolic class	χ^2^	DF	P > χ^2^	Parameter estimates full model		Preferred model
Survival of founders	Condensed tannins	3.32	3	0.34	Intercept	4.44 (0.69)***	Intercept
					GTmean	0.03 (0.14)^*n.s*^	
					Induced	0.42 (0.52)^*n.s*^	
					G*I	** − **0.30 (0.20)^*n.s*^	
	Total phenolics	0.56	3	0.91	Intercept	5.34 (1.46)***	Intercept
					GTmean	** − **0.12 (0.19)^*n.s*^	
					Induced	0.10 (0.19)^*n.s*^	
					G*I	** − **0.03 (0.05)^*n.s*^	
	Anthocyanins	0.65	1	0.42	Intercept	2.19 (3.60)^*n.s*^	Intercept
					Preformed	0.50 (0.63)^*n.s*^	
Fecundity	Condensed tannins	65.62	3	< 0.0001	Intercept	9.62 (0.84)***	**9.66 ± 0.84**^**<0.0001**^
					GTmean	** − **1.04 (0.14)***	** − 1.07 ± 0.12**^**<0.0001**^
					Induced	0.42 (0.52)^*n.s*^	**0.57 ± 0.22**^**0.017**^
		65.50	2	< 0.0001	G*I	− 0.30 (0.20)^*n.s*^	
	Total phenolics	64.62	3	< 0.0001	Intercept	15.21 (1.50)***	**14.59 ± 1.41**^**<0.0001**^
					GTmean	** − **0.90 (0.14)***	** − 0.88 ± 0.14**^**<0.0001**^
		64.31	2	< 0.0001	Induced	0.22 (0.18)^*n.s*^	** − 0.15 ± 0.06**^**0.018**^
					G*I	** − **0.02 (0.04)^*n.s*^	
	Anthocyanins	3.60	1	0.058	Intercept	6.07 (2.21)**	Intercept
					Preformed	** − **0.02 (0.02)^*n.s*^	

Aphid survival and fecundity. GLM models with Poisson distribution function and identity link estimating the general effect of phenolic classes on aphid survival and reproduction across experimental set-ups. The full model includes genotype mean of the phenolic class (GTmean), induced value of the same per sample, and the interaction. Preferred model indicates model after backward removal of insignificant effects. Model summary given in bold. Suggesting a lack of effect on founder survival but effects of both CTs and PTs on fecundity. Moreover, anthocyanins measured non-destructively before the start of the experiment explained neither survival nor fecundity of the aphids.

*n.s*. not significant.

Model significances***P < 0.0001;**P < 0.01;*P < 0.5.

### Weak relationships between specific phenolics

**Figure 4:**
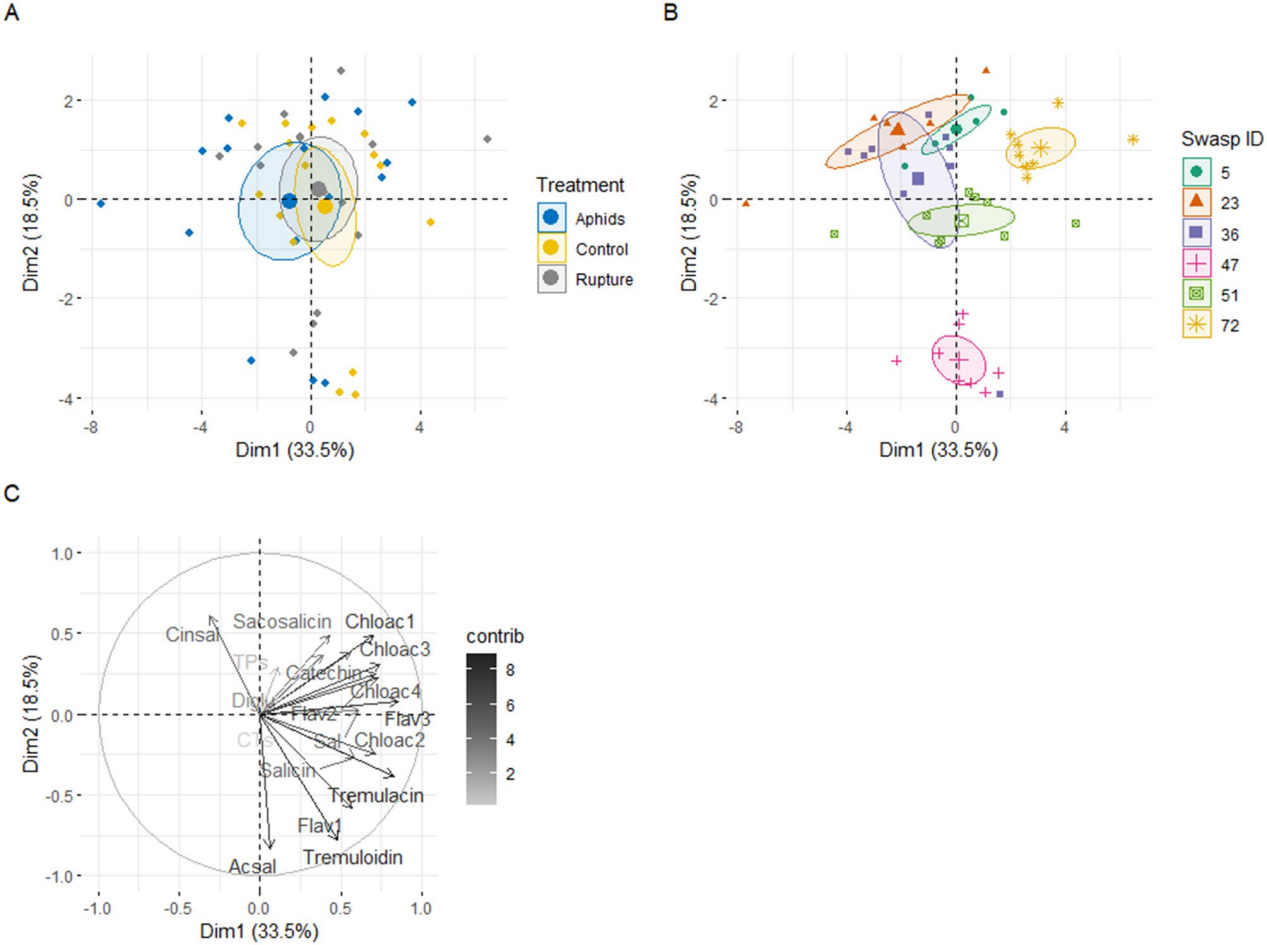
PCA ordination of foliar phenolic compounds (detected with LC/MS in peak area) and phenolic compound classes (total phenolics and condensed tannins in mg/g FW) in aspen plants shows that the association with (**A**) treatment, is less distinct that the association with (**B**) genotype. (**C**) PCA loadings highlight specific impact of the included phenolics, and suggest that *Tremuloidin, Tremulacin*, a Flavonoid (*Flav3*), and a Chlorogenic acid (*Chloac1*) belong to the stronger contributors to phenolic phenotypes. Additional compound abbreviations: Sal (Salicortin), Cinsal (Cinnamoyl Salicortin, Ac-sal (acetylsalicortin), Di-glu (diglucoside), Sa-co (salicoylsalicin), CTs (Condensed tannins), TPs (Total phenolics).

Detailed phenotyping of samples from Experiment 2 using LC–MS targeted chromatography focused on 16 phenolic compounds including catechin, six SPGs^12,49^, three flavonoids, and four chlorogenic acids. In a PCA model, 33.5% and 18.5% of the variance was explained by the first two ordinates, respectively (Fig. 4A–C, Scree plot in Supplementary Material File S3). In the PCA plot, genotype resulted in more apparent separation among samples (Fig. 4B) than treatment (Fig. 4A).

Linear models testing the relationship between TPs, soluble CTs, and specific phenolic classes such as flavan-3-ols and chlorogenic acids, provided some interesting insights (Table 3). Firstly, stronger relationships were detected for constitutive genotype means of TPs and CTs compared to post-induced values of the same. Secondly, the relationships were not strongly confined to sub-branches within the phenylpropanoid pathway. The strongest treatment effects were found for catechin, followed by chlorogenic acid3 and flavonoid3 and chlorogenic acid1 (Table 3). However, no relationship was detected for the SPGs: Salicin, Salicortin, and Tremulacin. Thirdly, compounds that were significantly related to CTs and TPs generally had opposing relationships with CT and TP. Thus, CTs negatively explained catechin levels whereas TPs positively explained the same (full model fits to be found in Supplementary Materials File S6).

**Table 3:**
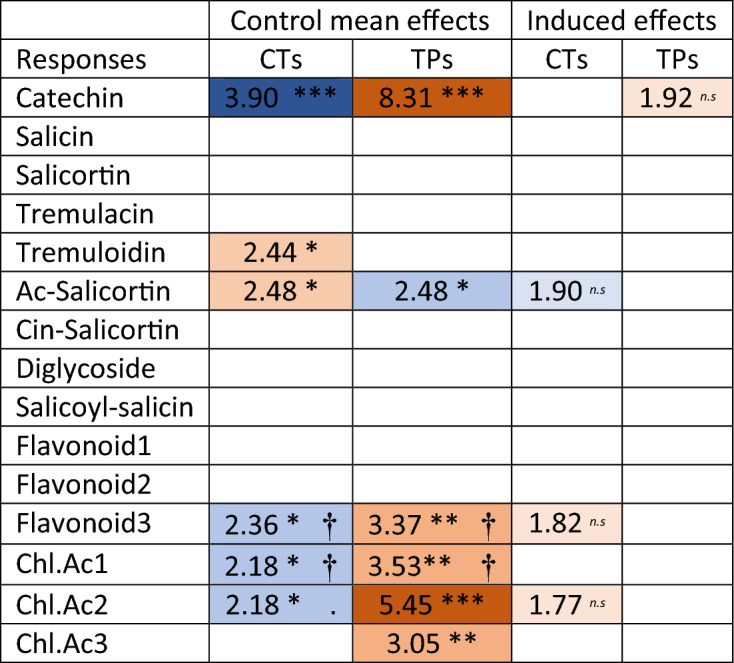
The importance of constitutive and induced CT and TP levels for induction of selected phenylpropanoids.

Treatment induced specific phenolic metabolites (17 phenolics obtained with use of LC/MS) were more often explained by genotype control means than by induced values of CTs and TPs. The two-factor general linear model, also included the effect of treatment (aphid and rupture), and full model summaries are listed in the Supplementary Materials File S6. LC/MS values were measured only in experiment 2 and the models were fitted to responses from plants under treatment, only. t-values for significant effects (df = 28, Significance levels:***P < 0.001;**P < 0.01;*P < 0.05; P < 0.1 = n.s.; P < 1 = no mark) are listed in the table, and colour codes indicate negative (blue) and positive (red) with intensity that correspond to significance value. ^†^Refers to situations where the model suggested treatment effects P < 0.1. Flavonoids 1, 2 and 3 (aromadendrin, Hesperetin 7-O-glucoside and aromadendrin-7-O-glucoside); Chl.Ac 1, 2 and 3 (5-*O*-(4-coumaroyl)-D-quinate, 5-O-caffeoyl-D-quinate, O-feruloylquinate).

### Phenolic induction showed compensatory phenolic dynamics

**Figure 5:**
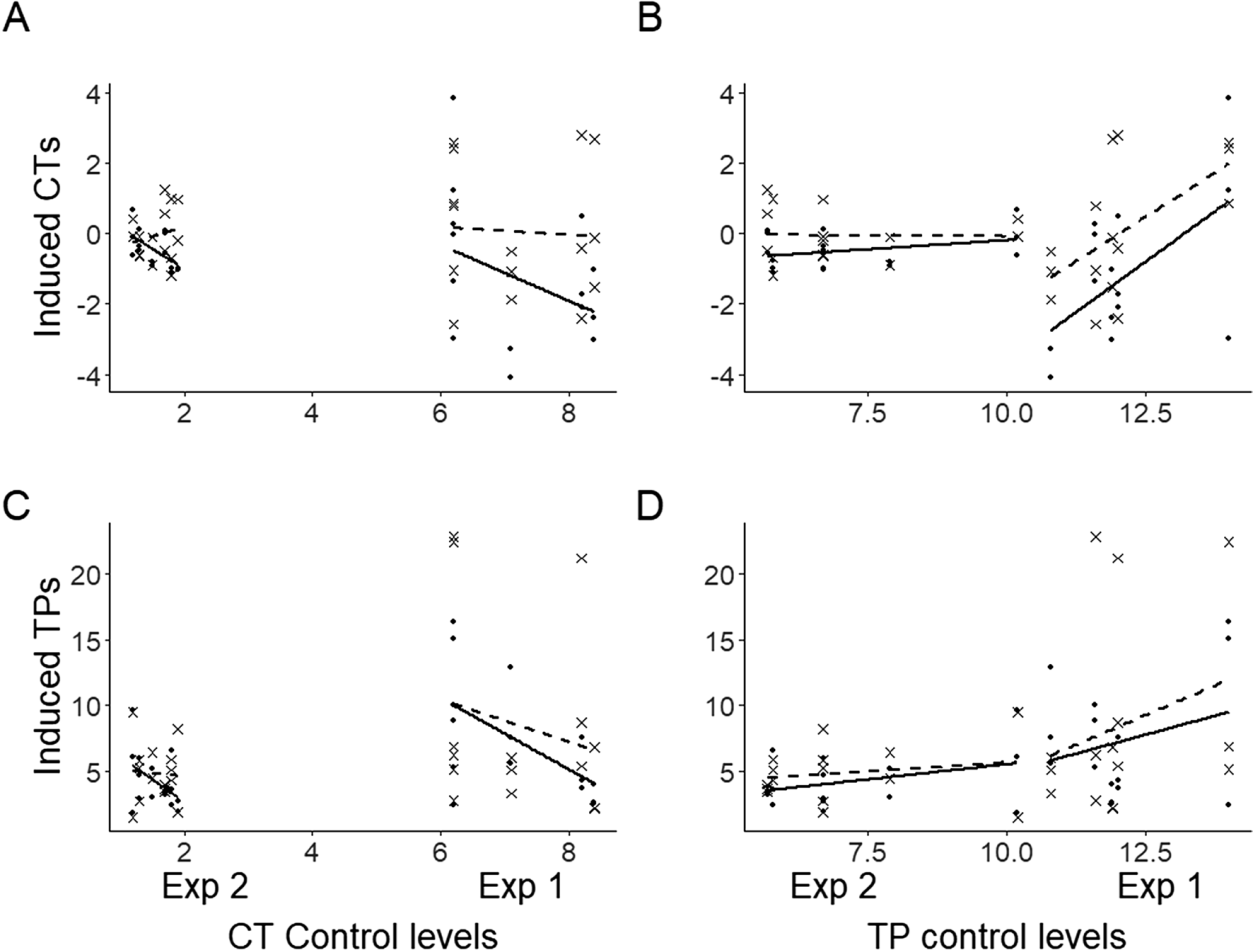
Genotype specific induced responses of condensed tannins and total phenolic glucosides in aspen leaves related to mean phenolic values of same genotype untreated controls. Full line = aphid infested plantlets; Stippled line = rupture damage. Test specifics are presented in Table S2.

Inducible relationships were explored for CTs and TPs (Fig. 5). The dynamic nature of CTs was obvious, with dry weight specific tannin levels in the second experiment dropping to one seventh of the concentrations measured for plants in the first experiment. By contrast, TPs only decreased by half between Experiments 1 and 2 (Supplementary Material File S3). Interestingly, as for the relationship with catechin, strong relationships were found between a genotype’s constitutive CT-values (as indicated by levels in the control plants), and inductive strengths measured for both CTs and TPs. This suggests that induction responses could be defined by control CT-levels and that a defence dynamic could be determined by biosynthetic dynamics, with correlative relationships between interacting compound pools, such as CTs and catechin units (Supplementary Material File S5, S7). Interestingly, the relationship between induced CTs and catechin differed between the two kinds of treatment in this study, supported by an interactive effect of treatment.

## Discussion

Growth and defence phenotypes allow us to study how plants allocate their resources under controlled stress conditions. We found genotype defined relationships between CT-levels in control plants and induction potential. *Chaitophorus* aphid reproduction and population size was mostly related to CT levels in control plants that positively correlated with induction of TPs and negatively with induction of CTs. However, TP levels (which include CTs) increased after treatment as did the induction of catechin. Although no single phenylpropanoid could explain aphid survival or fecundity, decreased CT and elevated catechin pools in aphid infested plants suggested that CT degradation could be involved in shaping resistance in aspen.

### Total phenolics best explained aphid resistance

Of all phenolic identities, TPs showed the strongest negative relationship with aphid infestation, and thus offered the best explanation for resistance (Fig. 4C). The Folin-Ciocalteu assay is a sensitive and reproducible method of assessing phenolic activity in plant tissues; however, despite its long history in that role, its reliability in quantifying phenolic compounds is questionable because the reagent not only measures phenols but also reacts with any reducing substance, and therefore measures the total reducing capacity of a sample^57,58^. Although not measured directly in this study, oxidative reactive chemicals such as H_2_O_2_ may indeed be a result of aphid damage in trees and herbs—for example, as oxidative flavon-3-ols are synthesised upon pathogen infection and aphid infestation as in Arabidopsis^39^ and Poplars^59^. Catechin also increased after treatment corresponding to a decrease in CTs, implying that catabolism of the polymer could be an indirect provider of resistance. Degradation studies have been conducted outside the living plant, for example in relation to nutrient turn-over in ecosystems^22^ and mammalian gut floras^57,60^, and although CTs-synthesis is well described^20,21,61^, we lack knowledge about potential plant enzymes that could perform such degradation.

### Relative CT-levels explained induced resistance properties

The present study highlights the dynamic nature of tannins, which varied sevenfold between experiments. The plants belonged to the same propagation event but the second batch for the second experiment was held in a greenhouse between the start of the first and the second experiment. November and December are two of the darkest months in northern Sweden during which daylight hours are reduced by ca 2.5 h down to 4.3 h; Our results suggest that both the age of the experimental plants and/or the greenhouse conditions may have impacted the replicated SwAsp clones between the two experiments, which were however conducted in climate chambers with a defined climate. From previous field studies we have reported tannin dynamics to depend on growth site^15^ and nutrient availability^13,22,62^. However, although tannin levels are dynamic, they are also synchronised between growth sites and years allowing for intra-specific division into high and low tannin-producing genotypes^14,19,44,63^. We found that genotypes richer in CTs reduced their tannin levels more strongly, which could be caused by a faster response dynamic and/or by responses of larger amplitude. Interestingly, while the induced tannin levels were not linked directly with properties associated with resistance to aphids, or with the induction of most specific phenylpropanoids and flavonoids, the strongest negative relationships were expressed for total phenolics and catechin units. This supports the hypothesis that tannins are involved in sink-strength dynamics, as suggested by Arnold and Schultz^64^. In fact, the present study suggests that: 1. the response norm and variability of tannins may be a key behind phenol induced resistance; 2. that induction may be translated into several pools of which catechin is one; 3. that a between CTs and TPs is dynamic and part of the induction act; and 4. that an individual high dynamic potential may be recognised as relatively higher levels of constitutive CTs at the population level. We therefore propose that it is the dynamic potential of aspen CTs (and potentially any Salicaceous tree species) that shapes tannin effects in plants rather than absolute ‘constitutive’ levels in any individual tree or population.

### Catechin induction negatively correlated with CTs and positively with TPs

Plants perceive damage differently depending on the damaging agent. The initial recognition of a threat is transduced molecularly, and then guided by hormonal cross-talks to optimize the defence response that matches the threat^65^. In the present study, aphids caused negative induction in CTs, not matched by the response to rupture. Evidence from targeted phenolic LC-analyses indicated that catechin is differently induced by the two treatments; increasing in response to aphids, and decreasing in response to rupture damage. Some other phenolic species such as Flavonoid3 and Chlorogenic acid1 also appeared to be differently affected by the treatments. Phenolic compounds often correlate positively within a biosynthetic sub-branch; however, specialised compound pathways are usually also highly reticulate^5,6,8,10^, potentially adding stochasticity to the resulting defence phenolic profile. Like other specialised products the diversity of phenylpropanoids is largely defined by the addition of moieties with less substrate specificity^12^, when compared to general metabolism^5,6^. To understand biosynthetic priorities and mechanisms behind such dynamics would require more detailed and targeted studies than presented here. The model status of *Populus*, makes aspen excellent for such studies. The molecular tools available for this genus allow for unique insights into the dynamics of phenolic defence chemicals besides a potential to uncover relationships with general metabolism and resistance properties. From our study here of six aspen genotypes, we conclude that aspens, as chemical factories, handle challenges individually according to themes of relatedness among phenolic pools and classes. Our study further supports the notion that CTs are good indicators of a genotypes relative growth and defence potential.

### Condensed tannins as defence compounds and beyond

CTs have long been suggested to have functions beyond defence. Arnold and Schultz (2002) for example suggested a relationship between CT loads and sink strength, as hybrid saplings exposed to damage enhanced their levels of cell wall invertase and considerably enhanced influx of carbon to CTs. For *P. tremula*, Ref.^35^ documented several regulatory nodes within the phenylpropanoid biosynthetic pathway that had effects on gene activity and CT production in low-CT trees under high soil nitrogen conditions, and on growth (or at least carbon allocation to other pathways) of high-CT trees under low soil nitrogen conditions. CTs as arbiters of abiotic stress tolerance was further suggested by Gourlay and Constabel^59^ who evidenced antioxidative properties of foliar CTs to protect photosystem II from damage and lessen leaf necrosis symptoms in over expressing and silenced MYC-hybrids. Young aspen trees produce more CTs than old trees^14^ and higher levels build up in field compared to greenhouse conditions. Thus, in addition to indirect resistance properties, as demonstrated in the present study, the CT pathway clearly appears to have other important functions related to growth and development.

## Supplementary Information

Supplementary Information.

## Data Availability

The original contributions presented in the study are included in the article/Supplementary Material, further inquiries can be directed to the corresponding author/s. Further, No licence no. or ethical requirements are needed to grow or transport SwAsp plantlets or trees within Sweden.
