# Supplementary material for: Compensatory phenolic induction dynamics in aspen after aphid infestation
Source: Sci Rep. 2022 Jun 10;12:9582. doi: 10.1038/s41598-022-13225-x (PMC9187625; doi:10.1038/s41598-022-13225-x)
Supplement: Supplementary file 1 — Supplementary Information. [file 41598_2022_13225_MOESM1_ESM.docx]

# Overview of supplemental materials

Supplementary Materail File S1: SwAsp genotype phenotypic characteristics ………………………………………………………………………..……………..

Supplementary Materail File S2: Means and standard deviations of response variables by experiment a-c) and genotype b-c) ………………….

Supplementary Materail File S3: Scree plot for Figure 3 ………………………………………………….……………………………………………………..………………

Supplementary Materail File S4: Linear relationships between phenolic compounds and aphid numbers (Exp 2) ………………………….........

Supplementary Materail File S5: Model fits: phenol class induction type relationships ……………………………………………………….…..……………..

Supplementary Materail File S6: Model fits: phenol class induction type - single compound relationships ……………………………..………………

Supplementary Materail File S7: Model fits: phenol class induction type relationship with catechin ……………………………………...……………… .

## Supplementary Material File S1

| 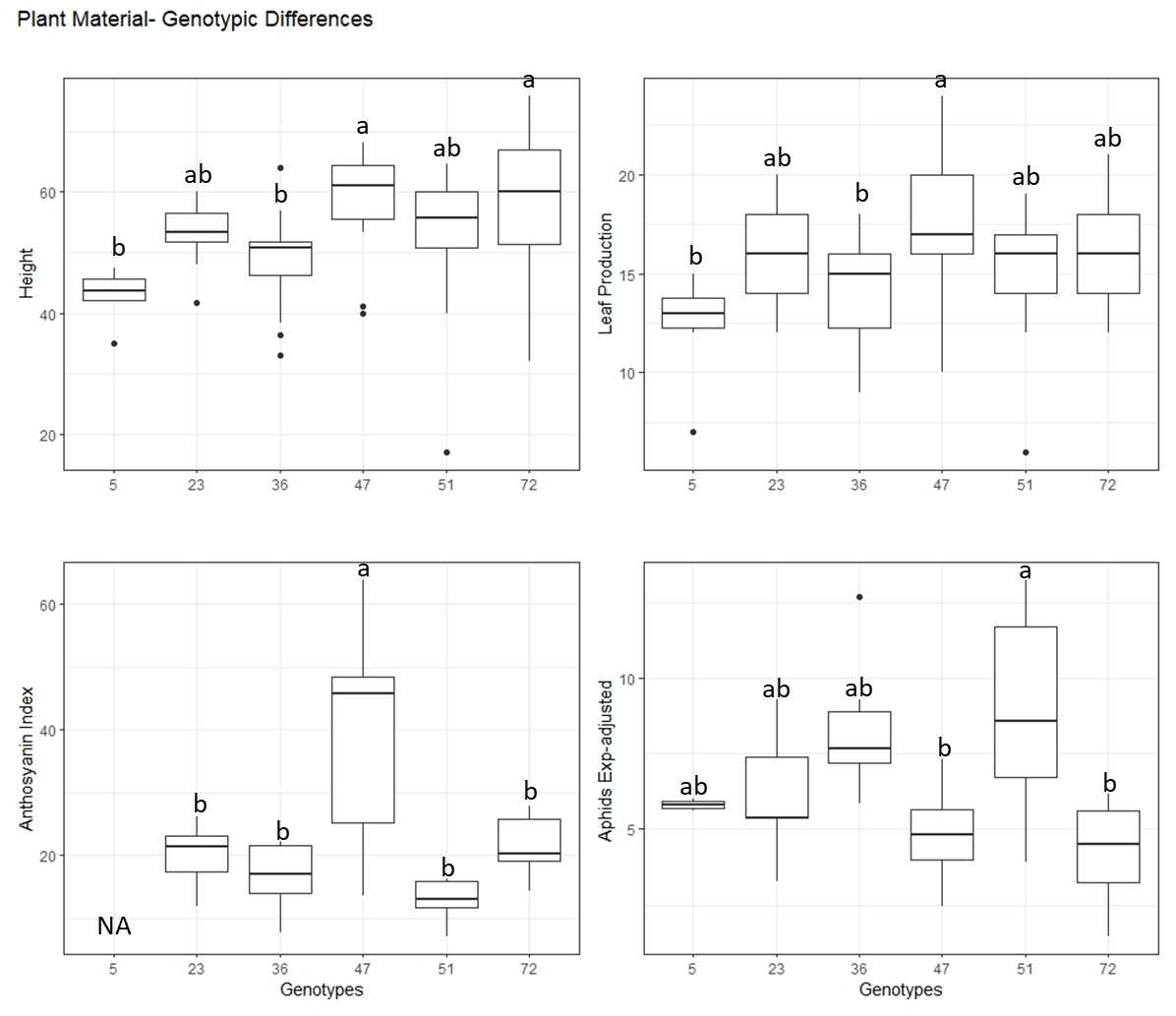 | Phenotyping six SwAsp plantlets suggested genotype differences in constitutive height, leaf production and Anthocyanin Index as well as in aphid susceptibility.  Height (in cm), leaf number, and Anthocyanin index in upper leaves (mean of six leaves only in experiment 1) were assessed before treatment start. Aphid numbers were normalised between two experiments to highlight genotypic differences.  Turkey’s Post-Hoc test detected SwAsp genotype differences for height (p < 0.00032***), leaf production (p < 0.0086**), anthocyanin levels (p < 5.36e^-07^ ***), and for aphid (adult and nymph) numbers (p < 0.0086**). Letter differences correspond to differences at p < 0.05. |
| --- | --- |

## Supplementary Material File S2

Means and standard deviations of response variables. Value assessments are described in detail in the methods section. ‘Total Aphids’ includes founders and offspring.

### S2b Mean ± sd by Exp & Treatment, Exp1

|  |  | ***Experiment 1*** | |  | ***Experiment 2*** | |
| --- | --- | --- | --- | --- | --- | --- |
|  | ***Aphid*** | ***Control*** | ***Rupture*** | ***Aphid*** | ***Control*** | ***Rupture*** |
| End Height (cm) | 71.03±10.9 | 77.87±10.42 | 68.83±10.59 | 110.88±13.86 | 113.63±15.56 | 121±20.41 |
| Leaf Production | 18.2±3.12 | 19.15±2.7 | 18.53±3.29 | 37.63±5.43 | 39.44±3.76 | 39.33±6.13 |
| CT mg/g FW | 5.93±1.99 | 7.07±2.4 | 7.26±2.06 | 1.05±0.42 | 1.57±0.64 | 1.52±0.81 |
| TP mg/g FW | 19.28±4.95 | 12.05±1.94 | 20.5±7.68 | 11.3±3.1 | 7.35±2.15 | 11.72±2.88 |
| Antho Up | 22.71±13.82 | 23.36±11.74 | 22.12±14.16 |  |  |  |
| Antho Down | 5.6±1.02 | 5.83±1.33 | 5.1±0.93 |  |  |  |
| Total Aphids | 13.67±6.79 |  |  | 45.75±18.3 |  |  |
|  |  |  |  |  |  |  |
| Ac-salicortin |  |  |  | 54.93±73.21 | 59.59±75.23 | 57.9±77.23 |
| Catechin |  |  |  | 1.97±2.3 | 2.48±2.72 | 2.39±2.33 |
| Chlorog Ac1 |  |  |  | 43.43±22.5 | 56.82±21.88 | 56.45±22.97 |
| Chlorog Ac2 |  |  |  | 4.5±3.82 | 5.71±4.24 | 4.5±3.63 |
| Chlorog Ac3 |  |  |  | 1.8±1.57 | 2.34±1.53 | 1.93±1.16 |
| Chlorog Ac4 |  |  |  | 7.71±6.57 | 7.82±3.42 | 9.48±9.94 |
| Cin-salicortin |  |  |  | 166.12±208.77 | 171.19±200.65 | 191.37±207.89 |
| Diglucoside |  |  |  | 98.59±30.1 | 110.58±16.12 | 109.16±14.99 |
| Flavonoid1 |  |  |  | 38.23±22.5 | 45.1±25.03 | 38.69±17.62 |
| Flavonoid2 |  |  |  | 23.27±11.33 | 27.74±10.08 | 28.24±7.66 |
| Flavonoid3 |  |  |  | 45.42±21.28 | 53.93±10.36 | 56.25±14.47 |
| Salicin |  |  |  | 133.16±42.93 | 150.8±25.38 | 151.24±24.09 |
| Salicortin |  |  |  | 378.05±177.67 | 455.89±108.3 | 391.1±149.84 |
| Salicoylsalicin |  |  |  | 1.23±0.69 | 1.33±0.45 | 1.24±0.65 |
| Tremulacin |  |  |  | 377.89±142.6 | 404.7±160.87 | 401.54±149.57 |
| Tremuloidin |  |  |  | 111.1±68.33 | 129.18±63.8 | 124.31±52.27 |

S2b Mean ± sd by Genet & Treatment, Exp1

| Genet |  |  |  |  | 23 |  |  | 36 |  |  | 47 |  |  | 51 |  |  | 72 |  |
| --- | --- | --- | --- | --- | --- | --- | --- | --- | --- | --- | --- | --- | --- | --- | --- | --- | --- | --- |
| N |  |  |  | 3 | 3 | 3 | 3 | 3 | 3 | 3 | 3 | 3 | 3 | 3 | 3 | 3 | 3 | 3 |
| ***Treatment*** |  |  |  | ***Aphid*** | ***Control*** | ***Rupture*** | ***Aphid*** | ***Control*** | ***Rupture*** | ***Aphid*** | ***Control*** | ***Rupture*** | ***Aphid*** | ***Control*** | ***Rupture*** | ***Aphid*** | ***Control*** | ***Rupture*** |
|  |  |  |  |  |  |  |  |  |  |  |  |  |  |  |  |  |  |  |
| End Height (cm) |  |  |  | 71.77±  7.01 | 68.15±  2.62 | 70.1±  0.95 | 65.27±  13.12 | 68.37±  2.85 | 56.43±  8.66 | 76.07±  11.15 | 84.00±  2.65 | 68.00±  10.54 | 72.43±  4.76 | 84.05±  0.07 | 70.83±  5.58 | 69.63±  19.21 | 83.6±  16.1 | 78.8±  13.36 |
|  |  |  |  |  |  |  |  |  |  |  |  |  |  |  |  |  |  |  |
| Leaf **Production** |  |  |  | 18.67±  1.53 | 17.5±  0.71 | 18.33±  1.53 | 16±  2.65 | 17.33±  2.31 | 17.67±  3.51 | 20.33±  3.79 | 20±  0 | 19.67±  6.03 | 18.33±  2.08 | 20±  0 | 17.67±  1.53 | 17.67±  5.03 | 20.67±  5.03 | 19.33±  4.16 |
|  |  |  |  |  |  |  |  |  |  |  |  |  |  |  |  |  |  |  |
| CT mg/g FW |  |  |  | 7.05±  1.4 | 8.16±  4.34 | 8.15±  2.61 | 3.57±  0.46 | 7.12±  0.73 | 5.96±  0.69 | 5.87±  0.85 | 6.24±  3.82 | 5.3±  1.68 | 6.27±  1.01 | 8.4±  2.38 | 8.75±  2.14 | 6.91±  3.44 | 6.22±  1.46 | 8.17±  0.95 |
|  |  |  |  |  |  |  |  |  |  |  |  |  |  |  |  |  |  |  |
| TP mg/g FW |  |  |  | 17.2±  2.05 | 12.02±  0.12 | 23.77±  8.33 | 19.41±  3.76 | 10.76±  2.92 | 15.54±  1.39 | 19.64±  2.45 | 11.59±  2.35 | 22.16±  10.74 | 14.94±  0.86 | 11.91±  0.57 | 15.62±  2.65 | 25.19±  7.7 | 13.93±  0.38 | 25.39±  9.51 |
|  |  |  |  |  |  |  |  |  |  |  |  |  |  |  |  |  |  |  |
| Antho Up |  |  |  | 17.54±  5.03 | 23.4±  0.8 | 20±  6.91 | 17.51±  3.82 | 15.81±  5.34 | 16.51±  7.71 | 43.23±  19.5 | 40.13±  13.98 | 39.9±  23.82 | 12.86±  2.98 | 14.73±  1.89 | 11.78±  4.31 | 22.4±  6.99 | 19.84±  0.76 | 22.42±  4.69 |
|  |  |  |  |  |  |  |  |  |  |  |  |  |  |  |  |  |  |  |
| Antho Down |  |  |  | 5.29±  0.86 | 4.58±  0.31 | 4.29±  0.85 | 5.89±  0.75 | 5.08±  0.7 | 5.54±  1.34 | 6.61±  0.74 | 7.59±  0.22 | 5.83±  0.99 | 4.81±  0.78 | 4.7±  0.85 | 5.16±  0.15 | 5.39±  1.42 | 6.4±  0.93 | 4.68±  0.48 |
|  |  |  |  |  |  |  |  |  |  |  |  |  |  |  |  |  |  |  |
| Total Aphids |  |  |  | 13.67±  4.62 |  |  | 19±  7 |  |  | 11.33±  3.51 |  |  | 17.33±  9.02 |  |  | 7±  4.58 |  |  |
|  |  |  |  |  |  |  |  |  |  |  |  |  |  |  |  |  |  |  |

### S2c Mean ± sd by Genet & Treatment, Exp2

| ***Genet*** | ***5*** |  |  | ***23*** |  |  | ***36*** |  |  | ***47*** |  |  | ***51*** |  |  | ***72*** |  |  |
| --- | --- | --- | --- | --- | --- | --- | --- | --- | --- | --- | --- | --- | --- | --- | --- | --- | --- | --- |
| N | 3 | 3 | 3 | 3 | 3 | 3 | 3 | 3 | 3 | 3 | 3 | 3 | 3 | 3 | 3 | 3 | 3 | 3 |
| ***Treatment*** | ***Aphid*** | ***Control*** | ***Rupture*** | ***Aphid*** | ***Control*** | ***Rupture*** | ***Aphid*** | ***Control*** | ***Rupture*** | ***Aphid*** | ***Control*** | ***Rupture*** | ***Aphid*** | ***Control*** | ***Rupture*** | ***Aphid*** | ***Control*** | ***Rupture*** |
| Final Height | 101±0 | 103±2.83 | 94±1.41 | 105.5±9.19 | 101.33±12.06 | 104.5±7.78 | 115.33±4.16 | 116±11.53 | 121±5 | 116.33±9.07 | 133±5.66 | 150±11.79 | 110.33±32.87 | 126.33±17.62 | 131.33±6.66 | 111.67±4.04 | 105±11.36 | 105.5±4.95 |
| Leaf Production | 33±1.41 | 37.5±2.12 | 31±2.83 | 37.5±6.36 | 38±2.65 | 38±4.24 | 38.33±2.08 | 36±1.73 | 40.33±4.04 | 41±7.21 | 42.5±3.54 | 48.33±1.53 | 36.67±10.02 | 43±3 | 39±1 | 37.67±1.53 | 40±5 | 34.5±4.95 |
| CT mg/g FW | 0.84±0.04 | 1.85±0.45 | 2.23±0.82 | 0.67±0.09 | 1.53±0.68 | 1.03±0.55 | 1.09±0.31 | 1.34±0.6 | 0.91±0.31 | 0.83±0.17 | 1.8±1.74 | 1.5±1.13 | 1.53±0.34 | 1.68±0.4 | 2.12±0.87 | 1.14±0.65 | 1.4±0.42 | 1.33±0.35 |
| TP mg/g FW | 9±0.61 | 6.7±2.03 | 11.73±4.47 | 11.96±1.5 | 7.86±1.14 | 13.3±1.4 | 11.16±1.54 | 6.67±0.81 | 11.23±1.65 | 10.02±2.1 | 5.83±1.32 | 10.9±0.8 | 9.13±0.31 | 5.65±0.81 | 9.35±0.26 | 16±3.93 | 10.69±2.06 | 15.64±5.65 |
| Total Aphids | 42.5±2.12 |  |  | 39±21.21 |  |  | 54.67±2.52 |  |  | 30.33±12.01 |  |  | 67.67±26.63 |  |  | 37±7.55 |  |  |
|  |  |  |  |  |  |  |  |  |  |  |  |  |  |  |  |  |  |  |
| Ac-Salicortin | 80±19.23 | 73.37±1.51 | 101.65±71.18 | 30.51±33.76 | 89.65±32.32 | 92.23±17.22 | 46.1±8.87 | 124.88±114.71 | 58.54±9.54 | 214.75±31.89 | 208.01±27.44 | 189.17±21.73 | 134.57±45.21 | 163.65±29.49 | 149.74±15.49 | 123.43±12.52 | 123.17±39.26 | 142.28±4.17 |
| Catechin | 132.65±13.15 | 126.67±11.29 | 120.14±8.76 | 62.09±71.25 | 102.95±16.94 | 106.8±12.44 | 117.22±13.52 | 123.61±20.52 | 118.84±25.1 | 81.69±14.07 | 108.3±4.8 | 96.08±9.02 | 90.89±12.92 | 107.71±17.26 | 112.39±11.59 | 106.18±2.74 | 98.85±6.52 | 100.8±2.23 |
| Chlorog Ac1 | 40.09±3.12 | 44.97±2.32 | 41.47±9.94 | 15.17±15.5 | 51.46±10.39 | 55.94±6.18 | 45.05±15.91 | 44.35±3.21 | 42.86±3.48 | 37.8±7.1 | 56.37±4.33 | 55.72±5.67 | 44.69±17.6 | 59.27±13.71 | 62.82±4.38 | 77.86±7.01 | 64.99±5.2 | 82.41±13.88 |
| Chlorog Ac2 | 58.32±6.06 | 51.27±1.1 | 41.38±8.96 | 30.76±34.18 | 68.1±4.17 | 66.1±4.92 | 38.98±6.27 | 30.49±25.63 | 45.53±12.01 | 25.48±15.54 | 38.26±3.65 | 32.62±9.07 | 40.22±23.43 | 66.01±20.26 | 70.35±3.75 | 67.55±24.13 | 78.78±6.58 | 93.14±28.8 |
| Chlorog Ac3 | 9.33±0.76 | 10.07±1.89 | 6.52±4.75 | 0.12±0.08 | 0.74±0.55 | 1.1±1.29 | 0.35±0.36 | 3±4.48 | 0.35±0.06 | 5.57±1.42 | 10.34±1.39 | 6.23±1.37 | 2.98±2.25 | 5.87±4.31 | 5.13±3.8 | 8.81±0.82 | 7.23±0.96 | 8.6±0.68 |
| Chlorog Ac4 | 2.35±0.78 | 1.94±0.29 | 1.9±0.38 | 0.84±0.83 | 1.17±1.02 | 1.99±0.58 | 0.97±0.12 | 1.4±0.44 | 0.96±0.79 | 0.75±0.16 | 1.88±0.16 | 0.87±0.23 | 1.33±1.36 | 3.23±2.02 | 2.47±0.19 | 4.4±1.22 | 4.15±1.55 | 4.11±0.49 |
| Cin-salicortin | 31.79±7.68 | 18.59±6.97 | 11.81±7.02 | 18.01±21.09 | 28.09±16.27 | 39.1±26.53 | 44.97±41.31 | 107±76.82 | 31.5±6.14 | 195.48±16.92 | 212.61±4.44 | 203.34±27.96 | 10.86±10.71 | 15.65±9.83 | 14.69±9.18 | 8.48±3.02 | 12.96±6.42 | 9±8.18 |
| Diglucoside | 2.55±0.71 | 1.74±0.58 | 1.22±0.01 | 0.73±0.79 | 1.51±0.5 | 1.77±1.42 | 1.19±0.43 | 1.57±0.29 | 1.58±0.77 | 1.08±0.26 | 0.95±0.16 | 0.71±0.08 | 0.74±0.32 | 0.81±0.27 | 0.85±0.08 | 1.38±0.45 | 1.42±0.29 | 1.6±0.4 |
| Flavonoid1 | 453.92±29.46 | 426.68±7.47 | 379.64±86.92 | 201.09±225.84 | 387.71±58.88 | 402.77±27.63 | 421.75±87.55 | 199.09±252.36 | 415.13±88.69 | 8.55±2.88 | 16.07±3.19 | 5.79±1.9 | 3.42±2.43 | 24.44±31.68 | 6.43±4.13 | 15.58±15.58 | 6.62±4.3 | 11.87±10.84 |
| Flavonoid2 | 29.83±12.81 | 22.45±4.01 | 13.39±5.68 | 8.87±8.08 | 25.75±9.9 | 32.17±15.66 | 36.78±17.18 | 67.34±34.12 | 42.76±23.47 | 69.61±17.64 | 79.62±3.37 | 47.37±7.43 | 23.8±13.48 | 39.57±17.54 | 35.12±9.09 | 333347.88±5.47 | 39.8±4.31 | 56.75±18.98 |
| Flavonoid3 | 35.29±5 | 34.73±4.98 | 23.87±9.74 | 8.4±7.96 | 27.66±9.65 | 30.82±8.82 | 21.77±13.57 | 25.19±3.71 | 20.76±7 | 18.94±5.12 | 17.3±22.25 | 26.31±4.54 | 21.53±12.77 | 33.67±12.63 | 35.96±2.29 | 32.75±2.94 | 26.74±3.61 | 32.57±7.31 |
| Salicin | 1.4±1.12 | 0.63±0.05 | 0.46±0.2 | 1.09±1.24 | 3.1±1.54 | 3.81±2.5 | 0.68±0.97 | 0.68±0.41 | 1.56±0.54 | 0.72±0.76 | 1.2±0.75 | 1.36±1.12 | 1.2±0.49 | 0.9±0.55 | 1.47±0.28 | 6.25±1.47 | 7.33±1.72 | 7.06±1.81 |
| Salicortin | 169.82±30.06 | 151.66±14.96 | 166.24±47.03 | 72.1±81.42 | 144.52±23.99 | 158.84±23.3 | 121.77±27.76 | 165.84±38.82 | 131.72±27.26 | 172.41±24.15 | 173.38±22.07 | 152.43±19.94 | 114.41±22.77 | 139.51±17.56 | 144.22±15.24 | 140.32±1.68 | 137.68±26.93 | 166.69±16.97 |
| Salicoylsalicin | 16.22±0.68 | 9.38±3.12 | 7.89±0.04 | 2.37±2.93 | 4.44±1.35 | 6.35±5.72 | 1.82±0.49 | 7.07±1.54 | 5.16±5.61 | 4.53±1.73 | 6.26±0.05 | 7.28±3.96 | 7.86±8.1 | 7.42±3.74 | 7.5±2.06 | 14.52±3.62 | 12.34±3.32 | 26.94±23.04 |
| Tremulacin | 522.38±28.35 | 427.93±27.47 | 329.33±188.17 | 115.79±98.63 | 452.57±45.92 | 289.86±298.66 | 314.94±275.13 | 527.63±100.96 | 510.29±159.87 | 421.77±59.96 | 494.15±18.34 | 391.44±27.48 | 369.38±187.87 | 362.12±213.49 | 368±45.4 | 484.73±35.41 | 474.39±76.59 | 409.44±244.87 |
| Tremuloidin | 367.21±77.68 | 311.02±68.57 | 261.36±22.69 | 124.68±139.54 | 185.99±137.71 | 253.88±48.54 | 281.17±69.25 | 382.21±115.62 | 277.83±71.79 | 463.72±42.7 | 562.11±12.53 | 434.33±27.62 | 404.59±54.36 | 506.8±143.83 | 557.76±16.34 | 538.04±36.46 | 501.34±54.25 | 591.42±143.5 |

## Supplementary Material File S3


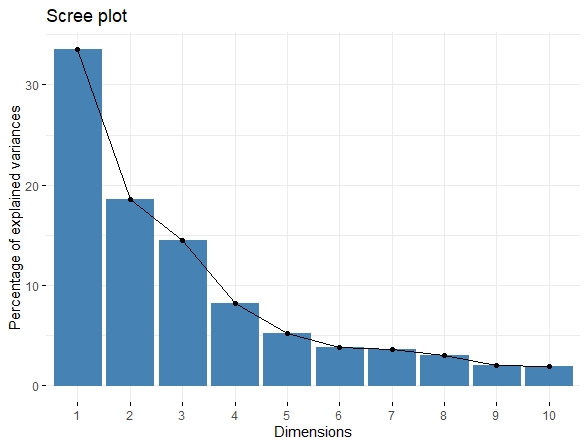


Scree plot indicating the explanatory power of the PCA, supporting Figure 3 based on Dimensions 1 and 2.

Supplementary Material File S4

| Aphid reproduction (the final number of adults and offspring) as affected by foliar phenolics. LC/MS only from the second experiment.    S4a) Salicinoid Phenolic Glycosides | 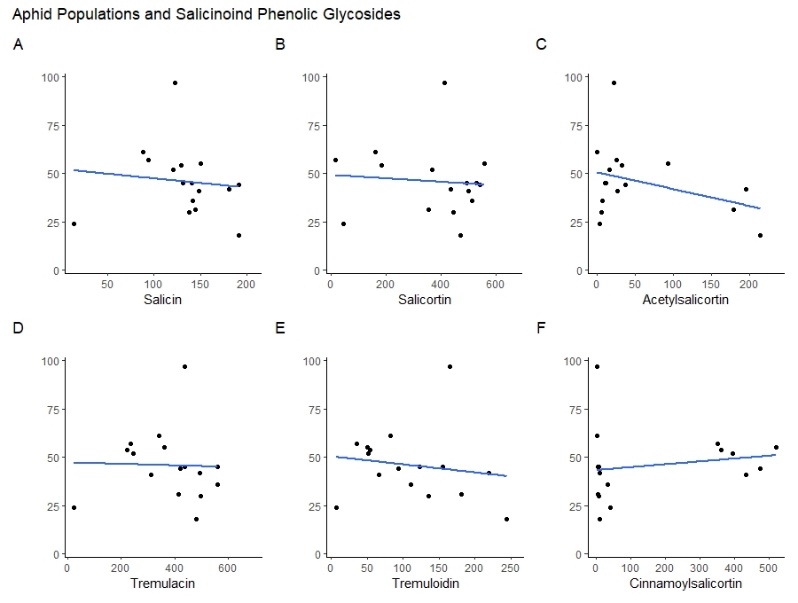 |
| --- | --- |
| S4b. Flavonoids | 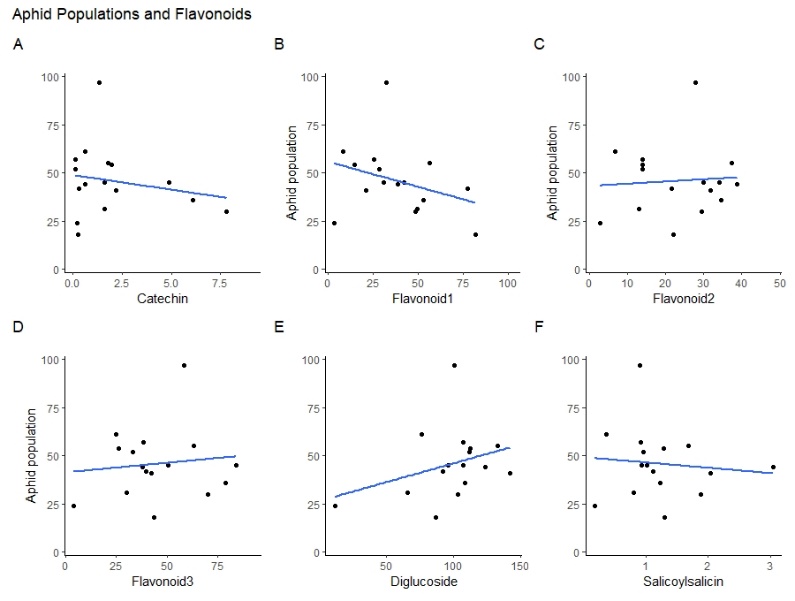 |
| S4c) Chlorogenic Acids | 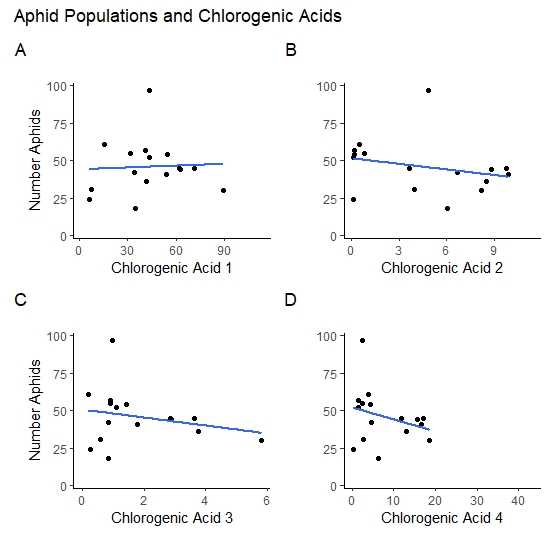 |

## Supplementary Material File S5

R-model: glmer(family = poisson, y ~ 1 + Constitutive (C) and Induced (I) + (1 + Genet|Exp)).

| **y** | **x** | **Features** | **Full model** | **Reduced Model I** | **Reduced Model II** |
| --- | --- | --- | --- | --- | --- |
| Total Aphids | **Condensed Tannins**    **Fixed effects:** | AIC  BIC  W (p-value)  R2-model  R2-fixed  Intercept  C  I  C*I | 284.72  320.57  0.83  0.93  0.50  4.32 (24.02) ***  -0.26 (-7.96) ***  0.18 (1.19) n.s.  -0.05 (-1.92) . | 323.20  331.80  0.12  0.92  0.20  3. 92 (38.16) ***  -0.10 (-4.69) ***  -0.06 (-1.72) . | 324.20  331.37  0.27  0.92  -0.12  3. 95 (3.95) ***  -0.08 (-2.72) ** |
|  | **Total Phenolics**  **Fixed effects:** | AIC  BIC  W (p-value)  R2-model  R2-fixed  Intercept  C  I  C*I | 304.99  315.03  0.73  0.92  0.48  4.20 (12.29) ***  -0.09 (-2.61) ***  0.02 (0.42) n.s.  -0.05 (0.45) n.s. | 304.29  312.89  0.78  0.92  0.52  4.49 (26.85) ***  -0.11 (-4.85) ***  -0.02 (-1.32) n.s. | 303.28  310.45  0.68  0.92  0.49  4.37 (20.38) ***  -0.11 (-4.26) *** |
| Nymphs | **Condensed Tannins**    **Fixed effects:** | AIC  BIC  W (p-value)  R2-model  R2-fixed  Intercept  C  I  C*I | 279.81  315.66  0.96  0.94  0.50  4.30 (24.02) ***  -0.33 (-7.96) ***  0.15 (1.19) n.s.  -0.05 (-1.92) . | 281.05  315.46  0.98  0.92  0.44  4.16 (16.97) ***  -0.29 (-7.57) ***  -0.13 (-2.03) * | 283.34  316.32  0.60  0.94  0.44  3. 92 (38.16) ***  -0.26 (-7.31) *** |
|  | **Total Phenolics**  **Fixed effects:** | AIC  BIC  W (p-value)  R2-model  R2-fixed  Intercept  C  I  C*I | 281.68  317.53  0.65  0.87  0.35  5.25 (8.61) ***  -0.24 (-4.81) ***  0.11 (1.45) n.s.  -0.01 (-1.79) . | 282.99  317.41  0.79  0.82  0.34  5.87 (10.54) ***  -0.29 (-6.88) ***  -0.02 (-1.41) n.s. | 282.66  315.65  0.76  0.91  0.35  3. 92 (9.70) ***  -0.30 (-6.08) *** |
| Adults | **Condensed Tannins**    **Fixed effects:** | AIC  BIC  W (p-value)  R2-model  R2-fixed  Intercept  C  I  C*I | 166.40  202.25  0.18  0.23  0.23  1.67 (8.70) ***  -0.05 (-1.10) n.s.  0.31 (1.21) n.s.  -0.06 (-1.49) n.s. | 166.51  200.92  0.49  0.09  0.07  4.16 (8.75) ***  -0.00 (-0.12) n.s.  -0.07 (0.35) n.s. | 164.99  197.98  0.40  0.00  -0.01  1.5 (38.16) ***  0.01 (0.17) n.s. |
|  | **Total Phenolics**  **Fixed effects:** | AIC  BIC  W (p-value)  R2-model  R2-fixed  Intercept  C  I  C*I | 168.91  204.76  0.27  -0.02  -0.03  0.00 (1.89) .  -0.00 (-0.16) n.s.  0.00 (0.00) n.s.  -0.00 (0.05) n.s. | 166.91  201.33  0.29  -0.01  -0.02  1.56 (10.54) ***  -0.01 (-0.20) n.s.  0.01 (0.31) n.s. | 165.02  198.00  0.38  0.92  0.20  1. 56 (4.06) ***  -0.004 (-0.10) n.s. |

## Supplementary Material File S6

Summary of two-factor linear models analysing for effects of phenolic class and treatment (aphid and rupture) on specific phenylpropanoids. Four models are tested per response including the phenolic factors: genet specific control means of CTs and TPs: CT-Ctrl and TP-Ctrl) and induced values of the same. Y- response values are 17 phenylpropanoids from Experiment 2: Catechin, Salicin, Salicortin, Tremulacin (Trem-cin), Tremuloidin (Trem-din), Acetylsalicortin (Ac-tin), Cinnamoylsalicotin (Cn-tin), Flavonoid1-3 (Flav1-3), Chlorogenic acid 1-4 (Ch_ac1-4), Diglycoside (Di_gly), Salicoylsalicin (Sac-cin). For each fit, distribution characteristics are listed with coefficient estimates (df=28) for Intercept, phenolic class (Phenol) and Treatment, and model fits (R^2^ and F_2,28_ ). t-values are listed for models and coefficient estimates with significance levels: 0 ‘***’ 0.001 ‘**’ 0.01 ‘*’ 0.05 ‘.’ 0.1 ‘ ’ 1. Colours correspond to significance and sign value (blue = negative / red = positive). For additional information see paper.

|  |  |  |  |  |  |  | Intercept |  |  | Phenol |  |  | Treatment |  |  | R^2^ | F_2,28_ |
| --- | --- | --- | --- | --- | --- | --- | --- | --- | --- | --- | --- | --- | --- | --- | --- | --- | --- |
|  |  | Min | 1Q | Median | 3Q | Max | Estimate | s.e. | t-value | Estimate | s.e. | t-value | Estimate | s.e. | t-value |  |  |
| Catechin | CT-Ctrl | -3.17 | -0.93 | -15.11 | 0.91 | 3.99 | 10.11 | 1.3 | 4.8 *** | -5.25 | 1.3 | -3.92 *** | 0.54 | 0.68 | 0.79 ^n.s.^ | 0.36 | 7.88 ** |
|  | TP-Ctrl | -2.67 | -0.92 | 0.09 | 0.88 | 2.09 | -6.78 | 1.1 | 8.3 *** | 1.22 | 0.2 | 8.31 *** | 0.67 | 0.46 | 1.46 ^n.s.^ | 0.71 | 34.98 *** |
|  | CT-Ind | -2.68 | -1.54 | -0.56 | 0.06 | 5.96 | 2.14 | 0.7 | 3.2 ** | 0.35 | 0.7 | 0.51 ^n.s.^ | 0.27 | 0.90 | 0.30 ^n.s.^ | 0.02 | 0.25 ^n.s.^ |
|  | TP-Ind | -3.33 | -0.99 | -0.57 | -0.10 | 4.98 | 0.42 | 0.98 | 0.4 ^n.s.^ | 0.37 | 0.2 | 1.92 · | 0.19 | 0.80 | 0.23 ^n.s.^ | 0.12 | 1.98 ^n.s.^ |
|  |  |  |  |  |  |  |  |  |  |  |  |  |  |  |  |  |  |
| Salicin | CT-Ctrl | -117.0 | -15.98 | -1.85 | 19.52 | 50.0 | 82.45 | 39.1 | 2.1 * | 32.72 | 24.6 | 1.3 ^n.s.^ | 17.32 | 12.47 | 1.39 ^n.s.^ | 0.12 | 1.94 ^n.s.^ |
|  | TP-Ctrl | -118.9 | -11.59 | 2.4 | 16.28 | 58.60 | 130.92 | 31.1 | 4.2 *** | 0.31 | 4.2 | 0.94 ^n.s.^ | 18.14 | 12.87 | 1.41 ^n.s.^ | 0.07 | 0.99 ^n.s.^ |
|  | CT-Ind | -119.5 | -12.20 | 3.01 | 16.22 | 57.33 | 132.48 | 10.33 | 12.8 *** | -1.38 | 10.5 | -0.13 ^n.s.^ | 18.69 | 13.65 | 0.90 ^n.s.^ | 0.02 | 0.99 ^n.s.^ |
|  | TP-Ind | -115.2 | -10.84 | -0.84 | 17.47 | 62.25 | 120.66 | 15.55 | 7.75 *** | 3.00 | 3.07 | 0.98 ^n.s.^ | 16.23 | 12.78 | 1.27  ^n.s.^ | 0.10 | 1.50 ^n.s.^ |
|  |  |  |  |  |  |  |  |  |  |  |  |  |  |  |  |  |  |
| Salicortin | CT-Ctrl | -368.07 | -61.45 | 33.93 | 112.82 | 268.6 | 420.2 | 188.9 | 2.2 * | -27.16 | 118.8 | -0.2 ^n.s.^ | 13.68 | 60.3 | 0.23 ^n.s.^ | 0.00 | 0.05 ^n.s.^ |
|  | TP-Ctrl | -359.13 | -55.89 | 33.26 | 113.75 | 277.20 | 343.87 | 145.7 | 2.4 * | 4.8 | 19.5 | 0.45 ^n.s.^ | 14.01 | 60.35 | 0.23 ^n.s.^ | 0.00 | 0.05 ^n.s.^ |
|  | CT-Ind | -361.26 | -60.01 | 26.23 | 119.24 | 274.72 | 376.86 | 48.48 | 7.8 *** | -2.4 | 49.3 | -0.05 ^n.s.^ | 14.10 | 64.05 | 0.22 ^n.s.^ | 0.00 | 0.02 ^n.s.^ |
|  | TP-Ind | -379.61 | -62.22 | 20.32 | 115.99 | 290.09 | 437.25 | 72.93 | 5.99 *** | -14.18 | 14.4 | -0.98 ^n.s^ | 21.80 | 59.93 | 0.36  ^n.s.^ | 0.04 | 0.51 ^n.s.^ |
|  |  |  |  |  |  |  |  |  |  |  |  |  |  |  |  |  |  |
| Trem-cin | CT-Ctrl | -352.6 | -120.4 | 40.88 | 110.1 | 285.7 | 401.25 | 167.4 | 2.4 * | -15.07 | 105.31 | -0.14 ^n.s.^ | 23.99 | 53.44 | 0.45 ^n.s.^ | 0.00 | -0.11 ^n.s.^ |
|  | TP-Ctrl | -363.9 | -121.9 | 36.36 | 103.46 | 239.29 | 263.48 | 127.2 | 2.07 *^.^ | 16.0 | 17.04 | 0.94 ^n.s^ | 26.9 | 52.7 | 0.51 ^n.s.^ | 0.04 | 0.54 ^n.s.^ |
|  | CT-Ind | -336.6 | -111.0 | 32.39 | 101.08 | 267.25 | 403.40 | 41.86 | 9.64 *** | 51.68 | 42.58 | 1.21 ^n.s.^ | 0.95 | 55.31 | 0.02 ^n.s.^ | 0.06 | 0.84 ^n.s.^ |
|  | TP-Ind | -357.0 | -121.9 | 34.68 | 111.83 | 276.5 | 396.7 | 65.57 | 6.05 *** | -4.51 | 12.95 | -0.35 ^n.s^ | 26.43 | 53.88 | 0.49 ^n.s.^ | 0.01 | 0.16 ^n.s^ |

File S5 page 2. Summary of linear models R-script = lm(formula = Y ~ X + TreatmentAR, data = aphidAR).

|  |  |  |  |  |  |  | Intercept |  |  | Phenol |  |  | Treatment |  |  | R^2^ | F_2,28_ |
| --- | --- | --- | --- | --- | --- | --- | --- | --- | --- | --- | --- | --- | --- | --- | --- | --- | --- |
|  |  | Min | 1Q | Median | 3Q | Max | Estimate | s.e. | t-value | Estimate | s.e. | t-value | Estimate | s.e. | t-value |  |  |
| Trem-din | CT-Ctrl | -104.94 | -41.91 | -4.28 | 46.05 | 108.51 | -40.5 | 63.6 | -0.64 ^n.s.^ | 97.81 | 40.03 | 2.44 * | 10.93 | 20.31 | 0.54 ^n.s.^ | 0.19 | 3.98 · |
|  | TP-Ctrl | -98.19 | -53.99 | 22.21 | 42.34 | 121.66 | 170.92 | 52.77 | 3.24 ** | -8.37 | 7.069 | -1.18 ^n.s.^ | 11.54 | 21.86 | 0.53 ^n.s.^ | 0.06 | 0.88 ^n.s.^ |
|  | CT-Ind | -104.56 | -56.26 | 7.50 | 37.88 | 132.76 | 110.94 | 17.98 | -0.02 ^n.s.^ | -0.32 | 18.28 | -0.02 ^n.s.^ | 13.36 | 23.75 | 0.56 ^n.s.^ | 0.01 | 0.18 ^n.s.^ |
|  | TP-Ind | -98.60 | -51.28 | -3.49 | 42.21 | 120.95 | 89.61 | 27.06 | 3.31 ** | 5.15 | 5.35 | 0.96 ^n.s.^ | 10.04 | 22.24 | 0.45 ^n.s.^ | 0.04 | 0.64 ^n.s.^ |
|  |  |  |  |  |  |  |  |  |  |  |  |  |  |  |  |  |  |
| Ac-tin | CT-Ctrl | -90.88 | -53.45 | -6.2 | 10. 51 | 148.47 | -134.0 | 78.06 | -1.7 · | 121.95 | 49.11 | 2.48 * | 0.12 | 24.92 | 0.00 ^n.s.^ | 0.18 | 3.09 · |
|  | TP-Ctrl | -83.35 | -46.83 | -26.15 | 20.49 | 153.18 | 197.90 | 60.25 | 3.29 ** | -19.99 | 8.07 | -2.48 * | -1.1 | 24.96 | -0.04 ^n.s.^ | 0.18 | 3.07 · |
|  | CT-Ind | -71.02 | -45.50 | 7.56 | 162.83 | 132.76 | 35.08 | 20.81 | 1.69 ^n.s.^ | - 40.23 | 21.17 | -1.9 · | 20.63 | 27.50 | 0.75 ^n.s.^ | 0.12 | 0.81 ^n.s.^ |
|  | TP-Ind | -59.22 | -44.52 | -33.57 | -8.01 | 176.62 | 50.89 | 33.82 | 1.51 ^n.s.^ | 0.97 | 6.68 | 0.15 ^n.s.^ | 2.37 | 27.80 | 0.09 ^n.s.^ | 0.001 | 0.02 ^n.s.^ |
|  |  |  |  |  |  |  |  |  |  |  |  |  |  |  |  |  |  |
| Cn-tin | CT-Ctrl | -221.1 | -165.8 | -134.1 | 194.3 | 340.5 | 307.2 | 237.4 | 1.29 ^n.s.^ | -90.99 | 149.34 | -0-61 ^n.s.^ | 27.38 | 75.78 | 0.36 ^n.s.^ | 0.02 | 0.24 ^n.s.^ |
|  | TP-Ctrl | -193.0 | -169.5 | -146.9 | 209.9 | 251.6 | 192.94 | 184.28 | 1.05 ^n.s.^ | -3.75 | 24.69 | -0.15 ^n.s.^ | 24.49 | 76.34 | 0.32 ^n.s.^ | 0.00 | 0.07 ^n.s.^ |
|  | CT-Ind | -272.3 | -167.3 | 102.7 | 186.3 | 399.7 | 128.09 | 59.59 | 2.15 * | -77.05 | 60.61 | -1.27 ^n.s.^ | 59.09 | 78.73 | 0.75 ^n.s.^ | 0.06 | 0.87 ^n.s.^ |
|  | TP-Ind | -190.8 | -171.8 | -146.3 | 211.2 | 357.2 | 175.33 | 93.74 | 1.87 · | -2.2 | 18.52 | -0.12 ^n.s.^ | 26.62 | 77.04 | 0.35 ^n.s.^ | 0.004 | 0.06 ^n.s.^ |
|  |  |  |  |  |  |  |  |  |  |  |  |  |  |  |  |  |  |
| Flav1 | CT-Ctrl | -35.65 | -14.57 | 0.36 | 12.47 | 46.58 | 55.99 | 23.02 | 2.4 * | -11.46 | 14.48 | -0.79 ^n.s.^ | 0.73 | 7.35 | 0.1 ^n.s.^ | 0.02 | 0.31 ^n.s.^ |
|  | TP-Ctrl | -35.96 | -14.61 | 0.78 | 12.04 | 45.31 | 29.74 | 17.88 | 1.67 ^n.s.^ | 1.19 | 2.39 | 0.50 ^n.s.^ | 0.71 | 7.41 | 0.1 ^n.s.^ | 0.009 | 0.13 ^n.s.^ |
|  | CT-Ind | -35.58 | -15.25 | -0.26 | 12.25 | 42.67 | 37.39 | 5.96 | 6.27 *** | -1.7 | 6.06 | -0.28 ^n.s.^ | 1.2 | 7.88 | 0.15 ^n.s.^ | 0.003 | 0.04 ^n.s.^ |
|  | TP-Ind | -35.1 | -15.19 | 0.63 | 11.76 | 43.77 | 38.33 | 9.13 | 4.2 *** | -0.02 | §.80 | -0.014 ^n.s.^ | 0.48 | 7.51 | 0.06 ^n.s.^ | 0.000 | 0.00 ^n.s.^ |
|  |  |  |  |  |  |  |  |  |  |  |  |  |  |  |  |  |  |
| Flav2 | CT-Ctrl | -20.54 | -9.39 | 0.37 | 7.96 | 15.85 | 24.57 | 11.16 | 2.20 * | -0.84 | 7.02 | -0.12 ^n.s.^ | 4.98 | 3.56 | 1.4 ^n.s.^ | 0.065 | 0.98 ^n.s.^ |
|  | TP-Ctrl | -21.51 | -8.55 | 0.93 | 7.39 | 16.16 | 13.65 | 8.4 | 1.63 ^n.s.^ | 1.35 | 1.13 | 1.2 ^n.s.^ | 5.24 | 3.48 | 1.51 ^n.s.^ | 0.11 | 1.74 ^n.s.^ |
|  | CT-Ind | -19.78 | 8.9 | 0.40 | 7.01 | 16.88 | 24.48 | 2.83 | 8.66 *** | 2.4 | 2.88 | 0.85 ^n.s.^ | 3.90 | 3.74 | 1.04 ^n.s.^ | 0.088 | 1.36 ^n.s.^ |
|  | TP-Ind | -21.3 | -8.7 | 0.54 | 7.03 | 15.42 | 26.20 | 4.3 | 6.05*** | -070 | 0.86 | -0.82 ^n.s.^ | 5.40 | 3.56 | 1.5 ^n.s.^ | 0.086 | 1.33 ^n.s.^ |

File S5 page 3. Summary of linear models R-script **=** lm(formula = Y ~ X + TreatmentAR, data = aphidAR).

|  |  |  |  |  |  |  | Intercept |  |  | Phenol |  |  | Treatment |  |  | R^2^ | F_2,28_ |
| --- | --- | --- | --- | --- | --- | --- | --- | --- | --- | --- | --- | --- | --- | --- | --- | --- | --- |
|  |  | Min | 1Q | Median | 3Q | Max | Estimate | s.e. | t-value | Estimate | s.e. | t-value | Estimate | s.e. | t-value |  |  |
| Flav3 | CT-Ctrl | -42.63 | -13.25 | 2.4 | 10.07 | 28.78 | 89.49 | 19.19 | 4.66*** | -28.43 | 12.07 | -2.36 * | 11.50 | 6.13 | 1.88 · | 0.24 | 4.34 * |
|  | TP-Ctrl | -45.84 | -8.91 | -0.35 | 12.48 | 22.07 | 1.3 | 13.67 | 0.92 ^n.s.^ | 6.17 | 1.83 | 3.37 ** | 12.09 | 5.66 | 2.14 * | 0.35 | 7.52 ** |
|  | CT-Ind | -38.41 | -11.31 | 1.99 | 10.14 | 40.01 | 50.07 | 5.10 | 9.83*** | 9.43 | 5.18 | 1.8 · | 6.70 | 6.73 | 0.99 ^n.s.^ | 0.18 | 3.12 · |
|  | TP-Ind | -40.21 | -10.82 | -3.65 | 9.05 | 38.88 | 41.74 | 8.199 | 5.09 *** | 0.88 | 1.62 | 0.55 ^n.s.^ | 10.29 | 6.74 | 1.53 ^n.s.^ | 0.10 | 1.47 ^n.s.^ |
|  |  |  |  |  |  |  |  |  |  |  |  |  |  |  |  |  |  |
| Di_gly | CT-Ctrl | -87.19 | -9.55 | 2.39 | 9.62 | 45.54 | 108.28 | 27.49 | 3.94 *** | -6.25 | 17.29 | -0.36 ^n.s.^ | 10.72 | 8.78 | 1.22 ^n.s.^ | 0.05 | 0.79 ^n.s.^ |
|  | TP-Ctrl | -87.11 | -10.46 | 2.49 | 11.11 | 43.49 | 96.43 | 21.25 | 4.54 *** | 0.30 | 2.85 | 0.11 ^n.s.^ | 10.64 | 8.80 | 1.21 ^n.s.^ | 0.05 | 0.73 ^n.s.^ |
|  | CT-Ind | -86.62 | -9.85 | 1.61 | 11.28 | 43.78 | 99.02 | 7.07 | 14.0 *** | 0.87 | 7.19 | 0.12 ^n.s.^ | 10.19 | 9.33 | 1.09 ^n.s.^ | 0.05 | 0.73 ^n.s.^ |
|  | TP-Ind | -87.12 | -9.71 | 1.97 | 11.30 | 42.87 | 99.47 | 10.81 | 9.02 *** | -0.21 | 2.14 | -0.1 ^n.s.^ | 10.70 | 8.88 | 1.2 ^n.s.^ | 0.05 | 0.73 ^n.s.^ |
|  |  |  |  |  |  |  |  |  |  |  |  |  |  |  |  |  |  |
| Ch_ac1 | CT-Ctrl | -38.49 | -13.50 | -0.35 | 15.92 | 44.69 | 94.72 | 24.10 | 3.93*** | -33.09 | 15.16 | -2.18 * | 13.80 | 7.69 | 1.79 · | 0.21 | 3.82 * |
|  | TP-Ctrl | -42.78 | -12.34 | 1.36 | 12.74 | 31.29 | -13.17 | 16.73 | -0.79 ^n.s.^ | 7.92 | 2.24 | 3.53 ** | 14.6 | 6.93 | 2.1 * | 0.36 | 8.01 ** |
|  | CT-Ind | -38.80 | -15.37 | -1.11 | 14.80 | 54.026 | 46.63 | 6.58 | 7.09 *** | 6.48 | 6.69 | 0.97 ^n.s.^ | 10.18 | 8.69 | 1.17 ^n.s.^ | 0.11 | 1.74 ^n.s.^ |
|  | TP-Ind | -36.06 | -13.54 | -0.17 | 13.09 | 59.29 | 40.58 | 10.21 | 3.98 *** | 0.68 | 2.02 | 0.34 ^n.s.^ | 12.6 | 8.39 | 1.2 ^n.s.^ | 0.08 | 1.29 ^n.s.^ |
|  |  |  |  |  |  |  |  |  |  |  |  |  |  |  |  |  |  |
| Ch_ac2 | CT-Ctrl | -4.49 | -3.284 | -1.31 | 3.76 | 6.32 | -0.25 | 4.18 | -0.06 ^n.s.^ | 3.07 | 2.62 | 1.17 ^n.s.^ | -0.07 | 1.33 | -0.05 ^n.s.^ | 0.46 | 0.68 ^n.s.^ |
|  | TP-Ctrl | -4.97 | -3.69 | 0.38 | 2.60 | 5.85 | -0.38 | 3.15 | -0.12 ^n.s.^ | 0.68 | 0.42 | 1.62 ^n.s.^ | 0.14 | 1.31 | 0.11 ^n.s.^ | 0.09 | 1.31 ^n.s.^ |
|  | CT-Ind | -4.68 | -3.67 | -0.76 | 3.52 | 5.93 | 5.07 | 1.08 | 4.7*** | 1.15 | 1.09 | 1.05 ^n.s.^ | -0.5 | 1.42 | -0.35 ^n.s.^ | 0.04 | 0.56 ^n.s.^ |
|  | TP-Ind | -4.62 | -4.02 | -0.62 | 3.83 | 5.54 | 5.16 | 1.67 | 3.09 ** | -0.16 | 0.33 | -0.48 ^n.s.^ | 0.1 | 1.37 | 0.07 ^n.s.^ | 0.008 | 0.11 ^n.s.^ |
|  |  |  |  |  |  |  |  |  |  |  |  |  |  |  |  |  |  |
| Ch_ac3 | CT-Ctrl | -2.10 | -0.80 | -0.45 | 0.95 | 3.31 | 4.91 | 1.47 | 3.35 ** | -2.01 | 0.92 | -2.18 * | 0.18 | 0.57 | 0.38 ^n.s.^ | 0.15 | 2.42 ^n.s.^ |
|  | TP-Ctrl | -2.01 | -0.61 | - 0.13 | 0.34 | 2.11 | -2.65 | .85 | -3.11 ** | 0.62 | 0.11 | 5.45 *** | 0.26 | 0.35 | 0.73 ^n.s.^ | 0.52 | 14.91 *** |
|  | CT-Ind | -2.03 | -0.89 | -0.19 | 0.89 | 3.2 | 2.14 | 0.39 | 5.54 *** | 0.69 | 0.39 | 1.77 · | - 0.17 | 0.51 | -0.34 ^n.s.^ | 0.10 | 1.60 ^n.s.^ |
|  | TP-Ind | -147 | -1.04 | -0.58 | 0.80 | 3.24 | 1.21 | 0.61 | 1.98 · | 0.14 | 0.12 | 1.18 ^n.s.^ | 0.05 | 0.50 | 0.09 ^n.s.^ | 0.05 | 0.73 ^n.s.^ |

File S5 page 4. Summary of linear models R-script = lm(formula = Y ~ X + TreatmentAR, data = aphidAR).

|  |  |  |  |  |  |  | Intercept |  |  | Phenol |  |  | Treatment |  |  | R^2^ | F_2,28_ |
| --- | --- | --- | --- | --- | --- | --- | --- | --- | --- | --- | --- | --- | --- | --- | --- | --- | --- |
|  |  | Min | 1Q | Median | 3Q | Max | Estimate | s.e. | t-value | Estimate | s.e. | t-value | Estimate | s.e. | t-value |  |  |
| Ch_ac4 | CT-Ctrl | -9.96 | -4.10 | -1.11 | 1.44 | 31.71 | 16.19 | 9.46 | 1.71 · | -5.47 | 5.95 | -0.92 ^n.s.^ | 1.89 | 3.02 | 0.6 ^n.s.^ | 0.04 | 0.59 ^n.s.^ |
|  | TP-Ctrl | -9.98 | -4.55 | -0.98 | 2.41 | 25,22 | -11.05 | -1.72 | -0.09 · | 2.62 | 0.86 | 3.05 ** | 2.30 | 2.66 | 0.87 ^n.s.^ | 0.26 | 4.88 * |
|  | CT-Ind | -8.44 | -5.80 | -2.74 | 2.96 | 33.18 | 8.32 | 2.45 | 3.39 ** | 1.22 | 2.49 | 0.49 ^n.s.^ | 1.22 | 3.24 | 0.38 ^n.s.^ | 0.02 | 0.29 ^n.s.^ |
|  | TP-Ind | -8.33 | -5.27 | -2.74 | 2.7 | 31.08 | 11.09 | 3.69 | 3.01 ** | -0.81 | 0.73 | -1.11 ^n.s.^ | 2.26 | 3.03 | 0.75 ^n.s.^ | 0.53 | 0.79 ^n.s.^ |
|  |  |  |  |  |  |  |  |  |  |  |  |  |  |  |  |  |  |
| Sac-cin | CT-Ctrl | -1.07 | -0.38 | -0.25 | 0.26 | 1.93 | 1.72 | 0.75 | 2.27 * | -0.32 | 0.48 | -0.67 ^n.s.^ | 0.16 | 0.24 | 0.07 ^n.s.^ | 0.016 | 2.22 ^n.s.^ |
|  | TP-Ctrl | - 1.14 | -0.35 | -0.19 | 0.25 | 1.87 | 0.40 | 0.57 | 0.70 ^n.s.^ | 0.12 | 0.08 | 1.54 ^n.s.^ | 0.03 | 0.23 | 0.14 ^n.s.^ | 0.08 | 1.91 ^n.s.^ |
|  | CT-Ind | -1.11 | -0.41 | -0.18 | 0.26 | 1.7 | 1.13 | 0.19 | 5.88 *** | -0.27 | 0.20 | -1.1 ^n.s.^ | 0.10 | 0.25 | 0.39 ^n.s.^ | 0.04 | 0.56 ^n.s.^ |
|  | TP-Ind | -1.07 | -0.44 | -0.19 | 0.30 | 1.80 | 1.28 | 0.30 | 4.3 *** | -0.01 | 0.06 | -0.19 ^n.s.^ | 0.02 | 0.25 | 0.06 ^n.s.^ | 0.00 | 0.02 ^n.s.^ |

## Supplementary Material File S7

Induced responses of Catechin as a function of constitutive and induced CTs and TPs. Full line = aphid infested plantlets; Stippled line = rupture damage. Test specifics are presented in table below.


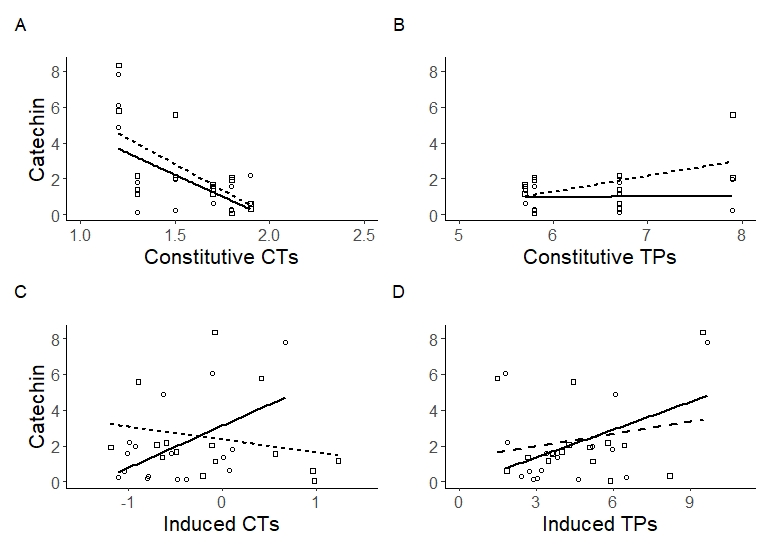


| 1. Effect of Constitutive CTs on Catechin | | | | | |
| --- | --- | --- | --- | --- | --- |
| *Model summary:* | | | | | |
| R^2^ = 0.36 | R^2^_adj_ = 0.29 |  |  |  |  |
| F-statistic _df=3,27_ | 5.12 | p-value: | 0.0062 | ** |  |
| Residual s.e. | 1.92 |  |  |  |  |
|  |  |  |  |  |  |
| *Coefficients:* | *Estimate* | *Std.* | *Error* | *T value* | *Pr(>\|t\|)* |
| (Intercept) | 9.46 | 2.93 | 3.23 | 0.0033 | ** |
| Constitutive CTs | -4.83 | 1.87 | -2.59 | 0.015 | * |
| Aphid vs Rupture | 1.96 | 4.32 | 0.45 | 0.65 |  |
| Interaction | -0.91 | 2.73 | -0.33 | 0.74 |  |
| 1. Effect of Constitutive TPs on Catechin | | | | | |
| *Model summary:* | | | | | |
| R^2^ = 0.72 | R^2^_adj_ = 0.69 |  |  |  |  |
| F-statistic _df=3,27_ | 22.76 | p-value: | 1.48E-07 | *** |  |
| Residual s.e. | 1.28 |  |  |  |  |
|  |  |  |  |  |  |
| *Coefficients:* | *Estimate* | *Std.* | *Error* | *T value* | *Pr(>\|t\|)* |
| (Intercept) | -6.3266 | 1.4525 | -4.356 | 0.000172 | *** |
| Constitutive TPs | 1.1604 | 0.1981 | 5.856 | 3.09E-06 | *** |
| Aphid vs Rupture | -0.3613 | 2.1686 | -0.167 | 0.86893 |  |
| Interaction | 0.1461 | 0.3012 | 0.485 | 0.631665 |  |
| 1. Effect of Induced CTs on Catechin | | | | | |
| *Model summary:* | | | | | |
| R^2^ = 0.36 | R^2^_adj_ = 0.09 |  |  |  |  |
| F-statistic _df=3,27_ | 1.94 | p-value: | 0.15 |  |  |
| Residual s.e. | 2.18 |  |  |  |  |
|  |  |  |  |  |  |
| *Coefficients:* | *Estimate* | *Std.* | *Error* | *T value* | *Pr(>\|t\|)* |
| (Intercept) | 3.14 | 0.77 | 4.09 | 0.00035 | *** |
| Induced CTs | 2.37 | 1.09 | 2.17 | 0.039 | * |
| Aphid vs Rupture | -0.79 | 0.95 | -0.83 | 0.415 |  |
| Interaction | -3.09 | 1.35 | -2.29 | 0.030 | * |
| 1. Effect of Induced TPs on Catechin | | | | | |
| *Model summary:* | | | | | |
| R^2^ = 0.14 | R^2^_adj_ = 0.05 |  |  |  |  |
| F-statistic _df=3,27_ | 1.48 | p-value: | 0.24 |  |  |
| Residual s.e. | 1.45 |  |  |  |  |
|  |  |  |  |  |  |
| *Coefficients:* | *Estimate* | *Std.* | *Error* | *T value* | *Pr(>\|t\|)* |
| (Intercept) | -0.19 | 1.29 | -0.15 | 0.88 |  |
| Induced TPs | 0.52 | 0.28 | 1.86 | 0.07 | . |
| Aphid vs Rupture | 1.48 | 1.93 | 0.77 | 0.45 |  |
| Interaction | -0.29 | 0.39 | -0.74 | 0.47 |  |

Signif.codes: 0‘***’ 0.001‘**’ 0.01‘*’ 0.05‘.’ 0.1‘ ’
